# Bodily maps of musical sensations across cultures

**DOI:** 10.1073/pnas.2308859121

**Published:** 2024-01-25

**Authors:** Vesa Putkinen, Xinqi Zhou, Xianyang Gan, Linyu Yang, Benjamin Becker, Mikko Sams, Lauri Nummenmaa

**Affiliations:** ^a^Turku PET Centre, University of Turku, Turku 20520, Finland; ^b^Turku Institute for Advanced Studies, Department of Psychology, University of Turku, Turku 20014, Finland; ^c^Institute of Brain and Psychological Sciences, Sichuan Normal University, Chengdu 610066, China; ^d^The Center of Psychosomatic Medicine, Sichuan Provincial Center for Mental Health, Sichuan Provincial People’s Hospital, University of Electronic Science and Technology of China, Chengdu 610072, China; ^e^MOE Key Laboratory for Neuroinformation, School of Life Science and Technology, University of Electronic Science and Technology of China, Chengdu 610054, China; ^f^College of Mathematics, Sichuan University, Chengdu 610064, China; ^g^State Key Laboratory of Brain and Cognitive Sciences, The University of Hong Kong, Hong Kong, China; ^h^Department of Psychology, The University of Hong Kong, Hong Kong, China; ^i^Department of Neuroscience and Biomedical Engineering, School of Science, Aalto University, Espoo 00076, Finland; ^j^Department of Psychology, University of Turku, Turku 20520, Finland

**Keywords:** music, emotion, body, cross-cultural

## Abstract

Emotions, bodily sensations and movement are integral parts of musical experiences. Yet, it remains unknown i) whether emotional connotations and structural features of music elicit discrete bodily sensations and ii) whether these sensations are culturally consistent. We addressed these questions in a cross-cultural study with Western (European and North American, n = 903) and East Asian (Chinese, n = 1035). We precented participants with silhouettes of human bodies and asked them to indicate the bodily regions whose activity they felt changing while listening to Western and Asian musical pieces with varying emotional and acoustic qualities. The resulting bodily sensation maps (BSMs) varied as a function of the emotional qualities of the songs, particularly in the limb, chest, and head regions. Music-induced emotions and corresponding BSMs were replicable across Western and East Asian subjects. The BSMs clustered similarly across cultures, and cluster structures were similar for BSMs and self-reports of emotional experience. The acoustic and structural features of music were consistently associated with the emotion ratings and music-induced bodily sensations across cultures. These results highlight the importance of subjective bodily experience in music-induced emotions and demonstrate consistent associations between musical features, music-induced emotions, and bodily sensations across distant cultures.

## Significance

Music is inherently linked with the body. Here, we investigated how music's emotional and structural aspects influence bodily sensations and whether these sensations are consistent across cultures. Bodily sensations evoked by music varied depending on its emotional qualities, and the music-induced bodily sensations and emotions were consistent across the tested cultures. Musical features also influenced the emotional experiences and bodily sensations consistently across cultures. These findings show that bodily feelings contribute to the elicitation and differentiation of music-induced emotions and suggest similar embodiment of music-induced emotions in geographically distant cultures. Music-induced emotions may transcend cultural boundaries due to cross-culturally shared links between musical features, bodily sensations, and emotions.

Music and the body are inherently linked (1). Spontaneous body movements such as head nodding and foot tapping are ubiquitous responses to music (2) that start to emerge already in infancy (3). Even in the absence of overt movement, mere exposure to music activates the sensory-motor regions of the brain (4), suggesting spontaneous motor planning, action simulation, or prediction (5, 6). Music induces changes in autonomic nervous system (ANS) activation indexed by heart rate, skin conductance, respiration, and body temperature (7–11). These changes are accompanied with endocrinological responses (for a review, see ref. 12) including changes in cortisol, oxytocin, and prolactin levels (13–15). Finally, listeners often report subjective sensations such as goose bumps, shivers down the spine and lumps in the throat, and changes in muscular tension, breathing, and heart rate while listening to emotionally engaging music (16–18). Music-induced bodily movements and physiological responses depend on the structural features and emotional content of music (19, 20), but little is known about how these factors influence the subjective bodily sensations induced by music and whether these associations generalize across different cultures.

Emotions support survival by guiding adaptive behavior (21). Music can induce a range of emotions (22, 23) and intense pleasure (24), but the potential evolutionary function and underlying mechanisms of music-induced emotions have remained elusive. Influential theoretical models assume that nonmusical emotions arise via the perception of changes in the internal state of the body, triggering corrective actions to return the body to homeostasis (25, 26). Although it is unclear whether music-induced emotions serve such homeostatic function, interoception and somatosensory feedback may constitute a key pathway also for the elicitation and differentiation of music-induced emotions (27), especially as music-induced emotions and body movement are closely intertwined (28). Accordingly, brain regions involved in autonomic regulation such as the insula and anterior cingulate cortex (ACC) (29) have been implicated in music-induced emotions (30). Moreover, neuroimaging studies indicate that emotion-inducing music modulates activity in somato-motor networks of the brain (31, 32) and pattern classification of fMRI data has shown that specific music-induced emotions can be decoded from the activity of the somatosensory and motor regions of the brain (33, 34). These findings suggest that different music-induced emotions might be associated with discrete patterns of bodily sensations similarly as has been established for prototypical, nonmusical emotions (35–37). This hypothesis however currently lacks direct empirical support.

Finally, music is a central part of social life in all cultures studied so far (38) and notable cross-cultural similarities exist in musical structure and music-related social practices (28, 38, 39). Dance is present in all studied cultures (38) indicating that body movement is an universal core component of music. This accords with the proposition that music’s ability to facilitate social affiliation through synchronized body movements (40) may have been one of the adaptive functions driving biological and cultural evolution of music (41). Akin to the evidence of cross-cultural recognition of emotions conveyed by vocalizations (42), similarities across distant cultures have also been documented in the recognition of emotions expressed in music (43) and emotions felt in response to music (22). These cross-cultural commonalities likely emerge from culturally universal links between acoustic features and affective evaluation (28, 44, 45). Together with the finding that bodily sensations associated with nonmusical emotions are consistent across a range of cultures (37), these results suggest that bodily sensations induced by emotional music may also show substantial cross-cultural similarities. Yet, this hypothesis remains to be empirically tested.

## Current Study

Here, we investigated how the topography of music-induced subjective bodily sensations varies according to the emotional characteristics and acoustic features of music. To test the generalizability of the results, we capitalized on a cross-cultural replication strategy with identical experiments in Western (United States and Western Europe) and East-Asian (China) participants using both Western and East Asian music (46). The focus on two cultures with diverging musical traditions and differences in various cultural dimensions and values (47) allowed us to determine the degree of cross-cultural generalization. We leveraged the fact that music can elicit a wide range of emotions that have been associated with distinct topographies of bodily sensations in studies using nonmusical stimuli (35–37). The participants listened to carefully validated happy, sad, tender, scary, aggressive, and danceable songs and reported their bodily sensations for each musical piece using the previously validated embody tool for bodily experience mapping (35). We employed music-information retrieval to map the acoustic and structural determinants of music-induced emotions and bodily sensations. The results revealed three topographically distinct clusters of bodily sensations, providing evidence for somatosensory representation of music-induced emotions. The music-induced bodily sensations and emotions were robustly replicable across the cultures and showed similar associations with the structural features of the music in both cultures. These results suggest similar embodiment of musical emotions across distant cultures that is mediated by shared emotional connotations of structural and acoustic features of music.

## Materials and Methods

### Stimuli and Participants.

The stimuli were 72 excerpts from 36 Western and 36 East Asian (Chinese) songs from six categories: happy, sad, scary, tender, aggressive, and danceable/groovy. The songs are listed in *SI Appendix*, Table S1. Western (N = 76) and East Asian (N = 23) subjects, who did not participate in the main experiment, respectively, rated initial sets of 108 Western and 78 East Asian song excerpts for happiness, sadness, scariness, tenderness, aggressiveness, and danceability. The affective dimensions used in the study were chosen because they constitute the most salient music-induced emotions and cover the four quadrants of the valence-arousal circumplex (48). Danceability was also included as it is directly related to music-induced body movements. To compile the initial song list, lab members and other local experts in Finland and China performed an extensive search in online music platform playlists to identify Western and Asian songs for each emotion category. We also consulted individuals specialized in specific subgenres, such as aggressive metal music, to extend and refine our list. Finally, we reviewed musical stimuli used in prior studies on music-induced emotions for additional examples. For each of the six categories, six Western and East Asian excerpts that received the highest ratings for the corresponding dimension were selected. A representative excerpt (mean duration of 34.7 s) was extracted from each song for the final stimulus set.

Data for the two main experiments were obtained online with the Gorilla experiment platform (app.gorilla.sc). In the first experiment (*SI Appendix*, Fig. S1), Western (n = 314, mean age = 32.4, SD = 9.0, 160 females) and East Asian (n = 518, mean age = 23.3, SD = 4.7, 343 females) participants rated the song excerpts for the aforementioned six dimensions (happiness, sadness, fear, tenderness, aggressiveness, and danceability). Four additional dimensions were included to i) quantify the affective arousal (energy, relaxation) and valence dimensions (liking, irritation) of the music. The subjects also rated how familiar they were with each song. On each trial, the participants were presented with a music clip and were asked to rate it for all 11 dimensions on a VAS scale (0 to 100) using sliders shown on the screen. In the second experiment, bodily sensations (*SI Appendix*, Fig. S1) evoked by the musical excerpts were measured with the emBODY tool (35) from an independent sample of Western (n = 589, mean age = 32.2, SD = 8.8, 275 females) and East Asian (n = 517, mean age = 23.1, SD = 4.3, 338 females) participants. All East Asian participants spoke Mandarin Chinese as their native language.

On each trial, the participants were presented with a music clip and a blank silhouette of a human body. They were asked to listen to the music and color the regions of the body whose activity they felt changing while listening to the piece. In both experiments, the participants were asked to complete 12 trials with one song excerpt randomly selected from each of six categories from the Western and East Asian stimulus sets. The Western participants were recruited from the United Kingdom and the United States of America via Prolific. The East Asian participants were recruited from China via campus mailing lists and advertisements. A systematic forward-backward translation of the instructions in the experiments was performed by X.Z., X.G., and B.B. The study protocol was approved by the Ethics Committees for Human Sciences at the University of Turku, Finland, and University of Electronic Science and Technology of China. All participants gave written informed consent online.

### Musical Feature Extraction.

Musical features were extracted with the MIRToolbox Version 1.7 (49) in MATLAB. We chose features that capture rhythmic (e.g., pulse clarity), tonal (e.g., key clarity), and timbral (e.g., spectral spread) aspects of the musical stimuli (50) and predict ratings of emotions expressed in music (51, 52). Full feature list is shown in *SI Appendix*, Table S2.

### Statistical Analysis.

Bodily sensation maps (BSMs) were first screened for anomalous response patterns (scribbling etc.), and responses outside the body area were masked out. To map where different emotional dimensions of music (happy, sad, scary, tender, aggressive, and danceable) are typically felt in the body, mass univariate *t* tests were used to compare pixelwise activations against zero for each category separately for the Western and East Asian subjects. This procedure resulted in six statistical summary maps (t-maps) per subject group, where pixel intensities reflect statistically significant bodily changes associated with each song category across subjects.

To quantify the similarity of the dimensional ratings between the Western and East Asian participants, we computed the mean for all dimensions within each category for both stimulus sets and computed the correlation between these mean ratings across the two cohorts. Linear mixed effects modeling was used to compare the dimensional ratings across the cohorts. To analyze the similarity between the dimensional ratings and BSMs, we generated distance matrices for the song-wise BSMs and dimensional ratings separately for the Western and East Asian participants and computed the correlations between the matrices with Mantel’s test. We also performed hierarchical clustering for the BSMs and ratings per category and stimulus set to further compare the structure of the responses in the two cohorts. To correlate the ratings for the 10 continuous dimensions with the BSMs, we first calculated the mean BSMs for each song and the mean ratings for each song and each dimension. To reduce the dimensionality of the statistical model, we then ran a principal component analysis (PCA) for the ratings and computed pixel-wise correlations between the song-wise BSMs and component scores. An analogous correlation analysis including PCA was conducted to correlate the musical features with the song-wise BSMs. See the appendixes for further details of the analyses and the code for reproducing them.

False discovery rate (FDR) correction (53) with an alpha level of 0.05 was applied to the statistical maps to control for false positives due to multiple comparisons in all analyses.

## Results

### Subjective Feelings.

**Fig. 1.**
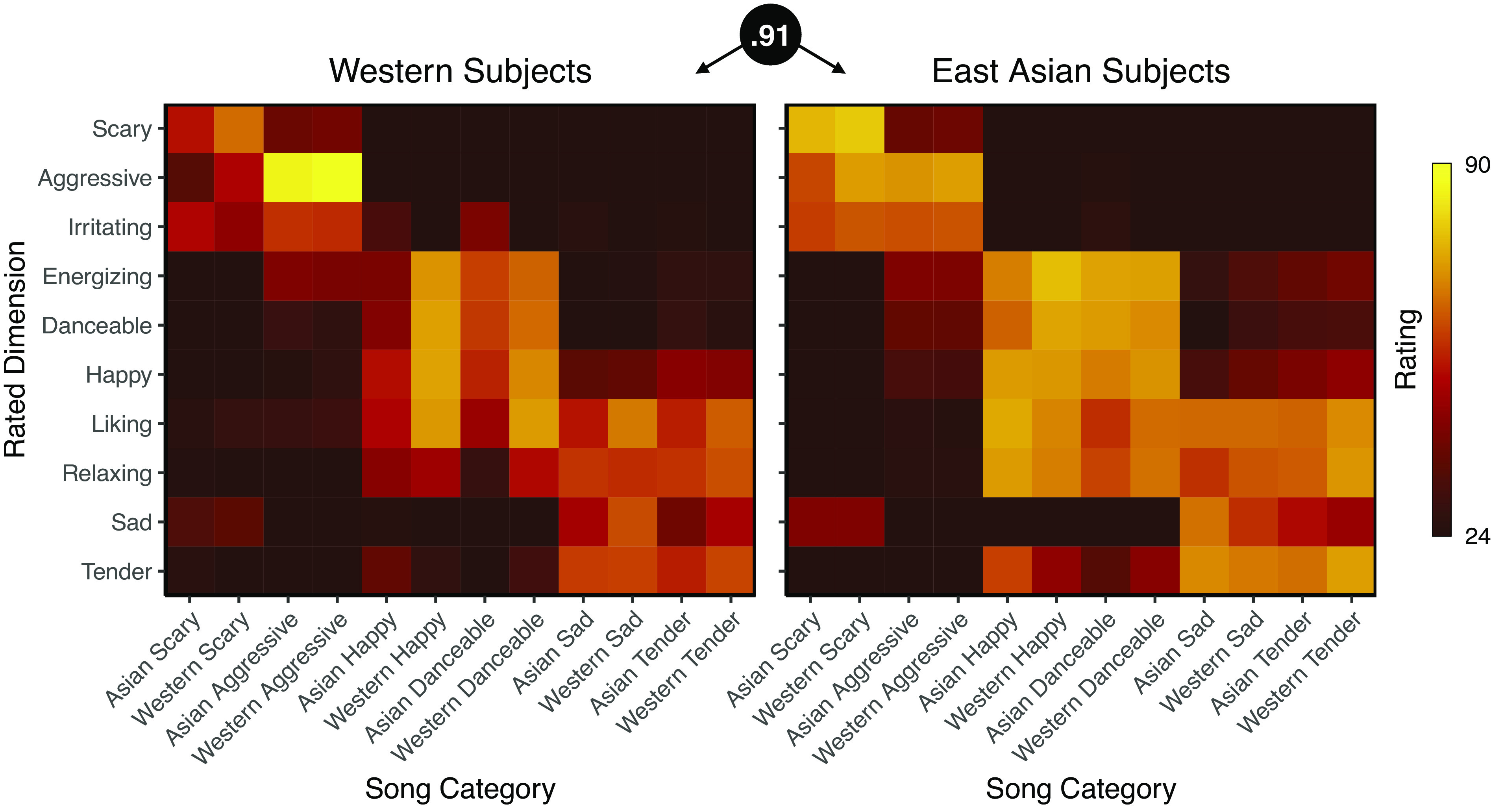
The mean ratings for the 10 target dimensions for each song category in Western and East Asian participants. The correlation between the matrices was 0.91 (*P* < 0.001).

The category-wise mean ratings for each dimension of the Western and East Asian participants were highly correlated (*r* = 0.91, *P* < 0.001), indicating that the musical stimuli induced consistent emotional experience across the two cultures (Fig. 1).

The clearest difference in the dimensional ratings between the cultures was that the Western subjects were less familiar with the East Asian songs than the Western songs, while the East Asian subjects were less familiar with the Western songs than the East Asian songs (dimension-wise comparisons between the cultures are reported and discussed in detail in *SI Appendix*). Thus, while the Western and East-Asian participants were not entirely culturally isolated from each other, the East-Asian participants reported significantly less familiarity with the Western songs, which are widely recognized in the West, and vice versa.

### Maps of Music-Induced Bodily Sensations.

**Fig. 2.**
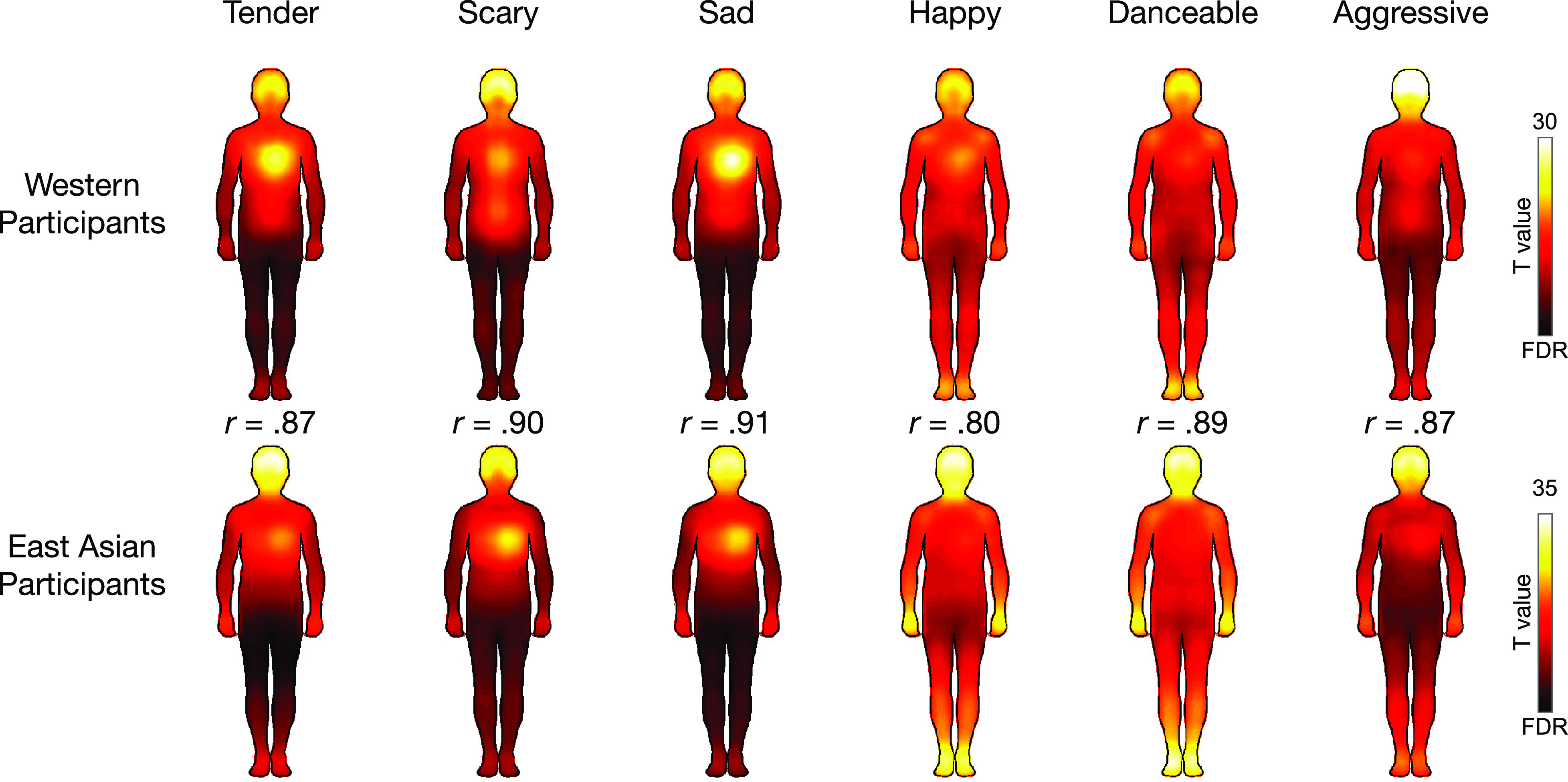
Topographies of bodily sensations evoked by each song category in Western and East Asian listeners. The maps show regions whose activation increased when listening to songs in each category (averaged over songs within each category, *P* < 0.05 FDR corrected). The correlation coefficients indicate the correlation between the BSMs of Western and East-Asian participants for each emotion.

Fig. 2 shows the emotion-wise BSMs for the Western and East Asian samples averaged across the Western and Asian songs (see *SI Appendix*, Fig. S2 for separate BSMs for Western and Asian songs and *SI Appendix*, Fig. S3 category-wise for SD maps). Tender and sad songs were felt primarily in the chest area and head, whereas scary songs also induced sensations in the gut area particularly in the Western participants. Happy and danceable songs led to widespread sensations throughout the body, particularly in the limbs. Aggressive music was also experienced saliently across the body and particularly in the head.

Despite the high overlap in the BSMs across the tested cultures some category-specific differences were also observed. The BSMs were compared across the two cultures using pixel-wise *t* tests with the null distribution generated through random permutations of group membership. These analyses revealed that East Asian participants reported more consistent activation mainly in the arms, legs, and head across the song categories. The Western participants showed more consistent activation in the chest for the tender and sad songs and in the abdomen region for the scary songs (*SI Appendix*, Fig. S4).

**Fig. 3.**
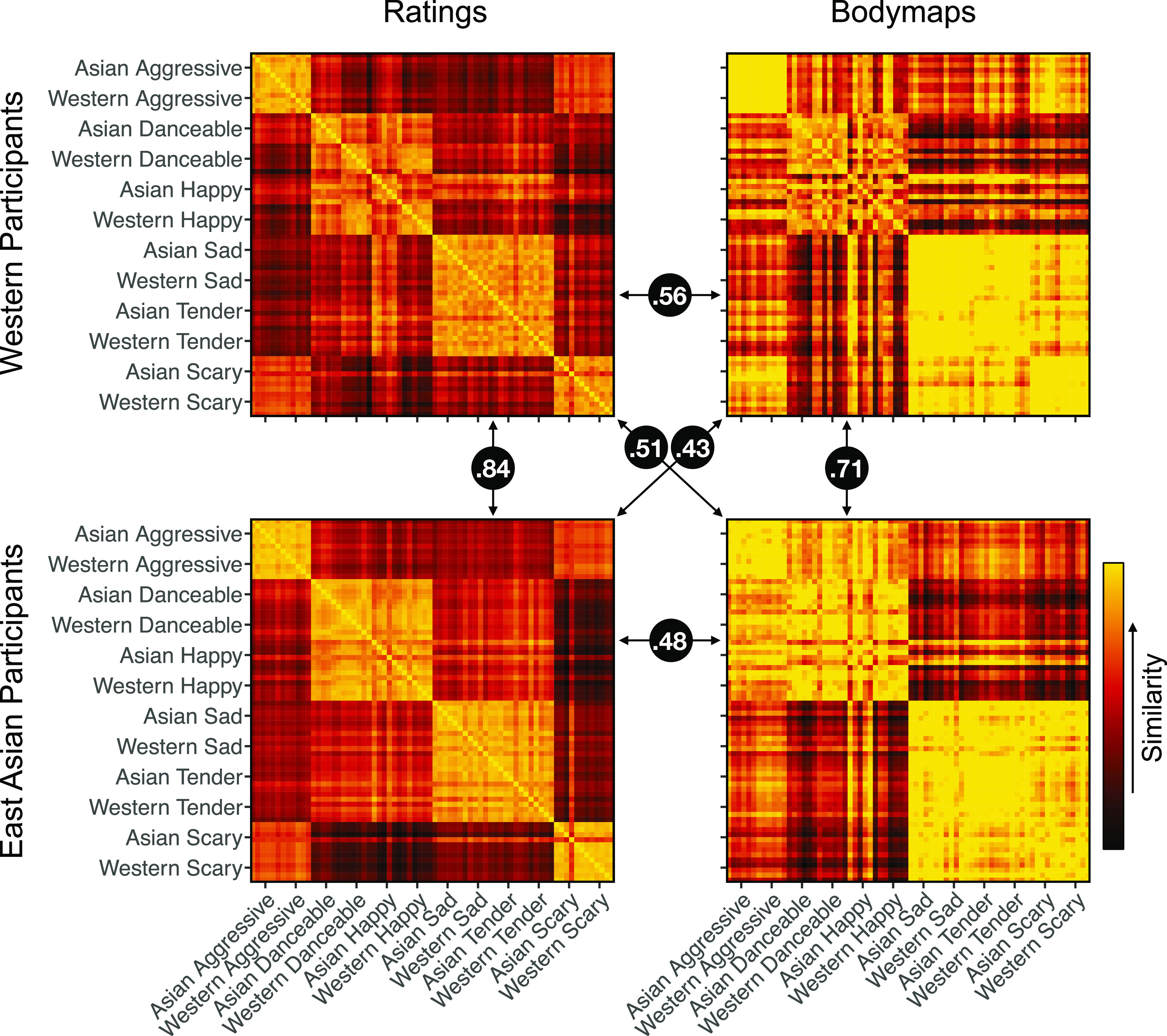
Similarity matrices for song-wise subjective ratings (*Left*) and bodily sensation maps (*Right*) for the Western (*Top*) and East Asian (*Bottom*) subjects. Black disks show correlations between matrices, all significant at *P* < 0.001 in Mantel’s test.

We next constructed similarity matrices (Euclidean distance) for the song-wise BSMs and corresponding ratings of emotion dimensions separately for the Western and East Asian participants (Fig. 3). The similarity matrices were correlated across Western and East Asian participants (BSMs: *r* = 0.71, *P* < 0.001; ratings: *r* = 0.84, *P* < .001, assessed using Mantel test with 1,000 permutations). The matrices were also significantly cross-correlated, indicating that similarity in emotion ratings predicted similarity in bodily sensations within as well as across the Western and East Asian subjects (Fig. 3 and *SI Appendix*, Fig. S7).

**Fig. 4.**
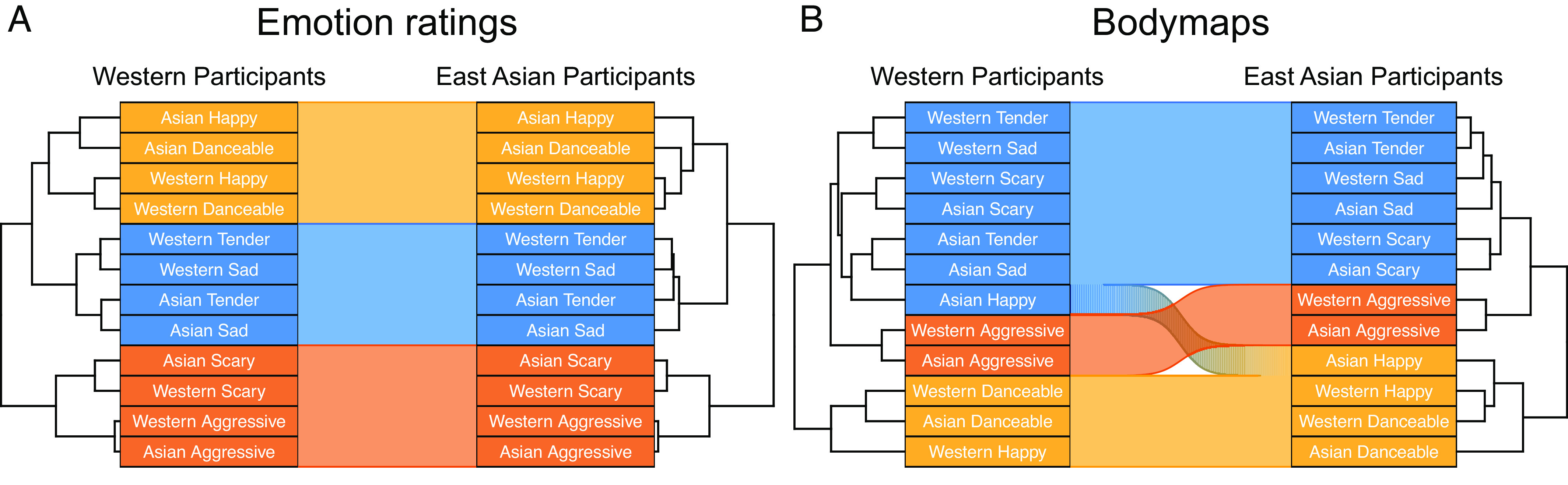
Hierarchical clustering of the emotion ratings (*A*) and BSMs (*B*) for the Western and East Asian listeners. A highly similar cluster structure was found in both samples.

Hierarchical clustering of the category-wise BSMs and ratings of emotional dimensions revealed a similar structure of the data in the Western and East Asian participants. For the ratings, happy and danceable songs formed one cluster, tender and sad another, and scary and aggressive songs a third cluster in both cultures (Fig. 4*A*). For the BSMs, tender, sad, and scary songs formed one cluster, danceable and happy another, and aggressive songs a third cluster. This cluster structure was found in both Western and East Asian participants with the exception that the Asian happy songs clustered with the tender, sad, and scary songs in the Western subjects (Fig. 4*B*).

**Fig. 5.**
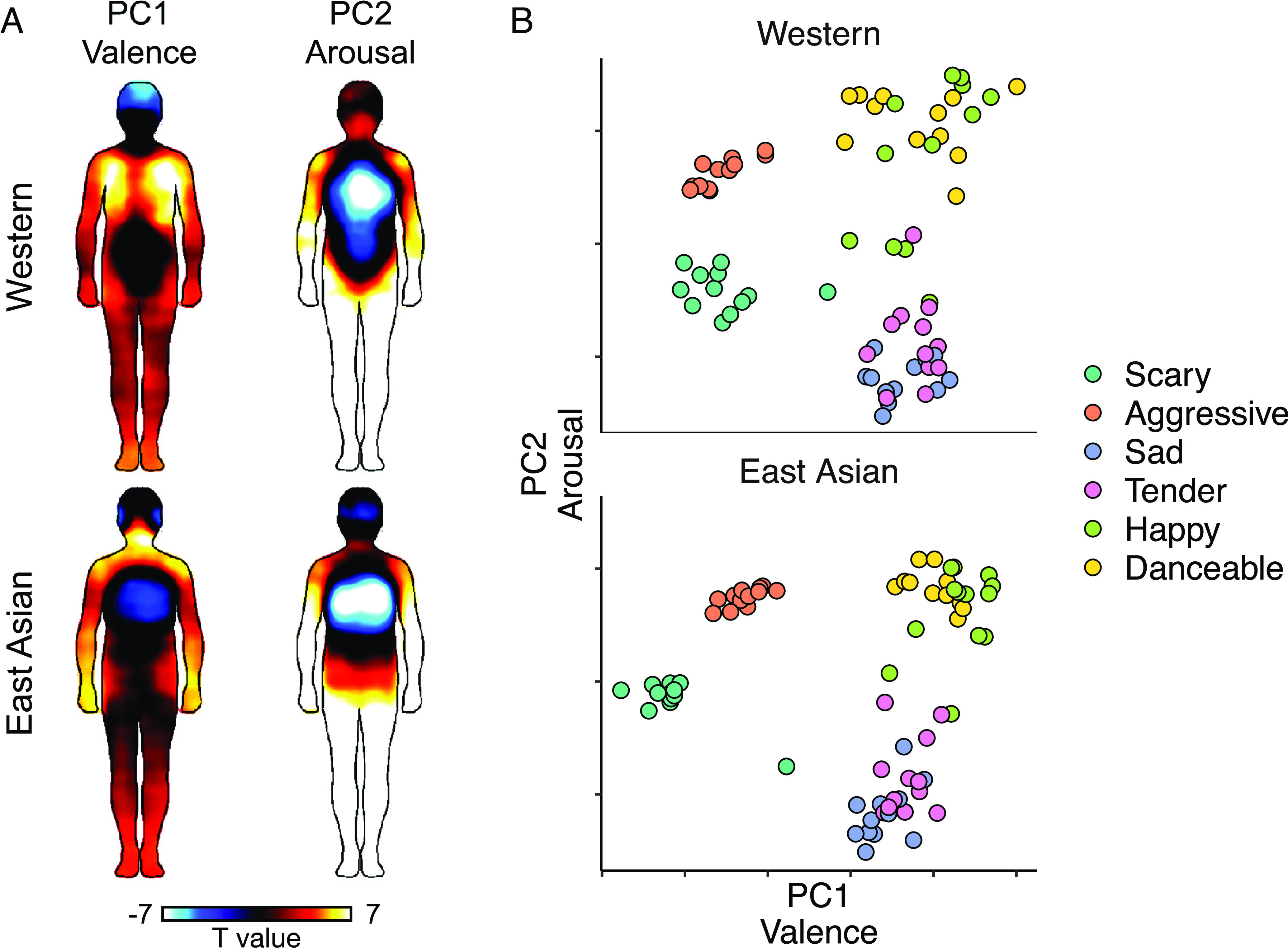
(*A*) BSMs for PC1 (Valence) and PC2 (Arousal) in Western and East Asian participants (*P* < 0.05 FDR corrected). (*B*) Song-wise PC scores.

We next ran a PCA for the dimensional emotion ratings and computed pixelwise correlations between the song-wise BSMs and the PC scores. Two principal components explained 92% and 97% of the variance in the dimensional emotion ratings in the Western and East Asian subjects, respectively (Fig. 5, the BSM for individual dimensions are shown in *SI Appendix*, Fig. S5). These components mapped roughly to the valence (pleasure-displeasure) and arousal (calmness-arousal) dimensions of emotion and showed a similar curvilinear relationship as has been previously obtained for valence and arousal ratings, e.g., pictorial stimuli (54, 55) (Fig. 5*B*). Both dimensions were mainly associated negatively with activations in the chest and positively with activation of the limbs in both subject groups (Fig. 5*A*). The PC scores were significantly correlated between the subject groups (PC1: *r* = 0.92, *P* < 0.001; PC2: *r* = 0.93, *P* < 0.001), indicating a similar component structure in the Western and East Asian subjects.

### Links between Acoustic Features and Emotional Ratings.

**Fig. 6.**
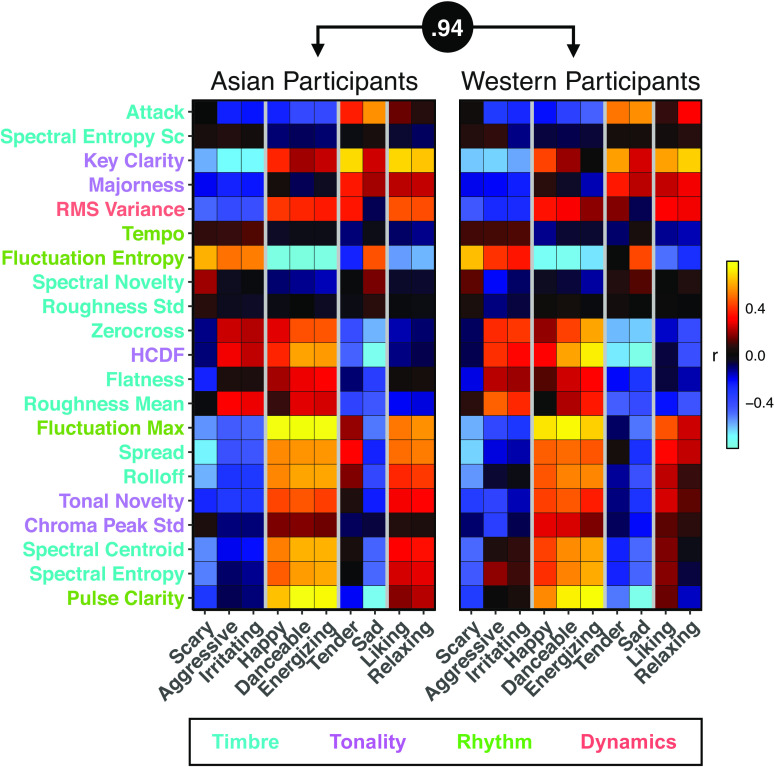
Correlations between musical features and dimensional emotion ratings in Western and East Asian participants. Similar correlation structures were obtained in both samples (*r* = 0.96). Colorbar indicates the strength and direction of the correlation. The gray vertical lines highlight dimensions with similar associations with the musical features. The order of musical features on the y is based on hierarchical clustering. The color of the feature name indicates whether the feature is related to the timbre (turquoise), tonality (purple), rhythm (green) or dynamics (red) of the music. See Table S2 for descriptions of the features.

The song-wise emotion dimension ratings and musical features had a similar correlation structure in Western and East Asian participants (correlation between correlation matrices in Fig. 6 = 0.94, *P* < 0.001), demonstrating that musical features are similarly predictive of music-induced emotions in both samples. The song-by-song similarity matrix of the musical features (*SI Appendix*, Fig. S7*A*) also correlated significantly with the corresponding BSM and rating similarity matrices in both samples (*SI Appendix*, Fig. S7*B*), indicating that the musical features, ratings, and BSMs displayed analogous similarity structures. These correlations were comparable across the samples, indicating that musical features were consistently associated with bodily sensations and emotional ratings in Western and East Asian listeners.

In both cultures, complex rhythms (high fluctuation entropy) and unclear key (low key clarity) were associated with scariness, aggressiveness, and irritation, while happiness, danceability, and energization showed essentially the opposite pattern and were also associated with a clear beat (high pulse clarity) (Fig. 6). Aggression and irritation were associated with roughness, a measure of sensory dissonance. Sadness and tenderness were associated with a clear key, little harmonic change (low HCDF), and timbres characterized by low frequencies, low attack times, and low roughness. Finally, liking and relaxation were most strongly associated with a bright timbre, a clear key, and a lack of rhythmic complexity (low fluctuation entropy).

### Links between Acoustic Features and Bodily Sensations.

**Fig. 7.**
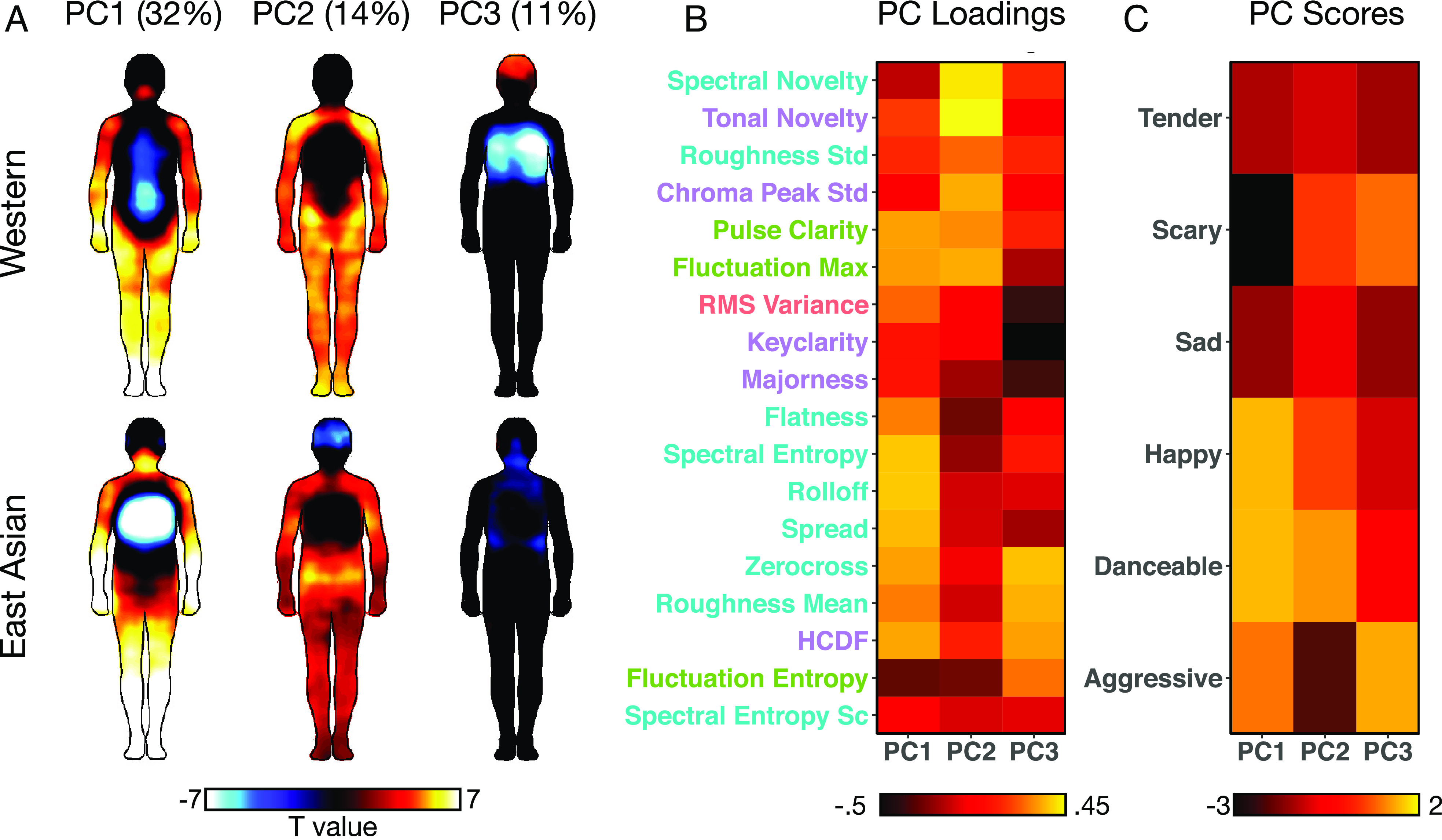
(*A*) Bodymaps for the musical feature PCs in Western and East Asian subjects (*P* < 0.05 FDR corrected). The percentages indicate the variance explained by each component. (*B*) Musical feature-wise PC loadings and (*C*) song category-wise PC scores pooled across the Western and East Asian songs. The color of the feature name indicates whether the feature is related to the timbre (turquoise), tonality (purple), rhythm (green) or dynamics (red) of the music. See Table S2 for descriptions of the features.

Because the musical features were correlated with each other, we ran a PCA to reduce the dimensionality of the data. We then computed pixel-wise correlations between the song-wise PC scores and BSMs for the first three components, which explained 58% of the variance (Fig. 7, see *SI Appendix*, Fig. S6 for BSMs for individual features). The first PC had the highest loading for features reflecting bright timbre (spectral roll-off, spectral entropy), strong pulse (pulse clarity), and frequency of harmonic change (HCDF). Happy and danceable songs had the highest scores for this PC and the scary songs the lowest (Fig. 7*B*). Accordingly, PC1 was positively associated with sensations in the limbs in both cultures and negatively associated with sensations in the lower abdomen and chest in the Western and East-Asian participants, respectively. The second PC had the highest loadings for features reflecting variation in the tonal and spectral profile (tonal novelty, spectral novelty). Danceable songs had the highest and aggressive songs had the lowest scores for this PC. Consequently, PC2 was also positively associated with activation in the limbs and hips. Finally, PC3 had the highest loadings for features reflecting distorted timbre and percussive sounds (zero crossing rate, roughness). Accordingly, the aggressive and scary songs had the highest scores for this PC and the sad and tender songs the lowest. PC3 also had a high negative loading from key clarity in line with the relative atonality of the aggressive and scary songs. In the Western subjects, PC3 was positively associated with activation in the head and negatively with activation in the chest, reflecting this PC’s high loadings for aggressive songs and low loadings for sad and tender songs. In the East Asian subjects, only some negative activation in the chest region not explained by PC1 reached significance. Thus, the BSMs for musical feature PCs showed cross-culturally consistent activation in the limbs but some differences were detected particularly in the abdomen and chest for PC1 and in the chest for PC3 (see *Discussion*).

## Discussion

Our main finding was that the topographies of music-induced bodily sensations vary according to the emotional and structural features of music while being consistent across participants and musical exemplars from Western and East Asian cultures. We observed close correspondence between music-induced subjective emotions and bodily sensations, suggesting that bodily responses might be a key pathway in the elicitation and differentiation of music-induced emotions (27). Given the cultural consistency of these effects, the results suggest similar embodiment of musical emotions across distant cultures and point toward a biological component in music-induced bodily sensations.

### Bodily Sensations and Emotions Evoked by Music Are Culturally Consistent.

Music-induced BSMs were highly consistent across the two cultures (*r* = 0.80 to 0.91, Fig. 3). Across both cultures, happy and danceable songs activated the arms, legs, and the head. In contrast, sad, tender, and scary songs activated mainly the chest and head regions while for the aggressive songs, the activation was mostly focused on the head. These cross-cultural similarities were corroborated by the hierarchical clustering of the BSMs which revealed this three-cluster structure in both cultures. The musical pieces were also rated consistently by the Western and East Asian subjects as indicated by the high correlation between the ratings across the groups (Figs. 1 and 3) and the similar structures obtained with hierarchical clustering of the ratings in both cultures (Fig. 4). Importantly, the subjective dimensional ratings and BSMs showed a broadly similar cluster structure and were correlated within and across cultures suggesting that music-induced subjective emotional experience reflects changes in internal bodily states (26).

In both cultures, two underlying dimensions reflecting valence and arousal explained considerable amount of variance in the ratings and these dimensions were also associated with distinct topographies of bodily sensations. This finding aligns with previous studies suggesting that valence and arousal capture substantial variance in subjective ratings for music-induced basic emotions (48) as well as ratings obtained with more music-specific emotional dimensions (56). However, a wider selection of musical material might have yielded a higher-dimensional structure (57). Indeed, there is compelling evidence that music can induce many nuanced categorical emotions in Western and Chinese listeners that are not reducible to arousal and valence (22).

The associations between the musical features, emotion dimension ratings and bodily sensations also showed remarkable correspondence between the Western and East Asian listeners, demonstrating that musical features are similarly predictive of music-induced emotions and bodily sensations in both cultures. For instance, happiness and danceability were positively associated with features reflecting a clear pulse and wide frequency spectrum (Fig. 6) which in turn were associated with the activation of the limbs in both cultures (Fig. 7). Cross-cultural similarities were found for lower-level musical features such as sensory dissonance as measured by roughness which was associated with dislike in both groups in line with previous cross-cultural studies on musical experiences (43, 58). The associations between low-level acoustic features of music and elicited emotions may mirror the function of these acoustic cues in communicating emotions through human vocalizations (59). Importantly, we also found cross-cultural consistencies in the association of higher-order structural features such as key clarity and tonal novelty with emotion ratings (Fig. 6). These results indicate that emotions can be communicated through music across diverse cultures because of shared emotional connotations of specific acoustic and structural cues. These findings suggest that specific musical features may be associated with an embodied, somatomotor representation (e.g., due to rhythmic entrainment) which shape emotional experience irrespective of culture.

Cultural differences have been observed in the rhythm perception and emotional connotations of dissonance and harmony (58–62), as well as in extra-musical factors like functions motivating music-listening (63) and narratives generated in response to music (64) that may influence emotional and bodily responses to music. Although above-chance level identification of happiness, sadness, and fear in western music has been reported in Mafa listeners, their performance was comparatively lower than that of Canadian listeners (43), indicating a substantial influence of cultural learning. While the music-induced BSMs and dimensional emotion ratings were consistent across the cultures examined, we also observed some differences. East Asian subjects reported more consistent sensations in the head and limb regions for most song categories as well as, more sensations in the chest region for the scary songs than the Western subjects (*SI Appendix*, Fig. S4). Western subjects, in contrast, reported more consistent sensations in the stomach region for the scary and sad songs. These differences were also observed in the lower-order bodily representations in the principal component analysis (valence; PC1, Arousal; PC2). When PCs were derived for the musical feature-dependent BSMs, the clearest cross-cultural differences were observed for the third PC (PC3) explaining the least overall amount of variance in the data. Notably, these differences were mainly driven by more consistent responses in one culture (East Asian) within regions where the other (Western) also showed significant responses, thus indicative of differences in sensation intensity or consistency across participants rather than topography across cultures (see *SI Appendix* for further discussion). Also, we stress that these differences are relatively small when compared to the robust overall similarities in the BSMs and self-reported emotions across cultures (correlations ranging from 0.80 to 0.91).

### Musical Motions and Emotions.

Our results conclusively show that music induces strong subjective bodily feelings which are correlated with music-induced emotional experience (Fig. 3). These bodily experiences could reflect skeletomuscular activity and changes in the autonomic nervous system state and may contribute to the embodied experience of musical emotions. For example, high emotional arousal induced by happy, danceable, and aggressive music is reflected in elevated activity of the limbs (Fig. 5). This may reflect the typical movements such music tends to induce in the listener: motion capture data indicates that spontaneous moving to high-arousal music is characterized by accelerated hand, foot, and head movements (65). The elevated activation of the chest area for the sad, tender, and scary songs, in turn, may reflect subjective perception of changes in heart rate and respiration. The sad and tender songs were rated high on relaxation and low on energization suggesting that these songs induced a decrease in heart rate (66) and slower breathing (11). The scary songs also received low ratings for energization and had relatively low scores for the arousal-related PC (Fig. 5*B*) which may explain why the BSMs to these songs were rather similar to those of the sad and tender songs, although their emotional impact was otherwise distinct. The scary songs were also disliked which has been associated with slower heart rate and respiration in previous studies (11, 66).

The predictive coding model (PCM) proposes that music perception and music-induced movement are based on Bayesian processes, wherein the brain seeks to minimize hierarchical prediction errors (6): listeners make predictions about unfolding musical events that are passed down to resolve prediction errors. The errors then propagate upward in the hierarchy to refine the predictions. The urge to move to a steady “beat” arises when the listener attempts to enforce the representation of musical pulse by making sensorimotor predictions about body movements, even without physically executing them (5, 6). This happens particularly when the music is syncopated and harmonically complex leading to a pleasurable sense of “groove” and urge to move (67–69). A cross-cultural work has established an inverted U-shape relationship between the level of syncopation and the urge to move (70) suggesting that prediction errors and precision might mediate bodily responses to music across diverse cultures.

In sum, our results suggest that emotion-specific bodily experience arising from primed movements and changes in physiological body states contribute to the elicitation and differentiation of music-induced emotions. Although bodily feedback may not be necessary for subjective experience of emotion (e.g., ref. 71; for a discussion, see ref. 72), our results are in line with models highlighting the role of interoception in emotion (25, 26). Somatosensory and motor cortices also encode representations of musical emotions, as indicated by studies showing that music-induced emotions can be reliably decoded from activity in these regions (33, 34) possibly due to their involvement in representing bodily states associated with different music-induced emotions. It should be noted that the current data cannot establish a causal relationship between music-induced bodily sensations and emotions. Nevertheless, our findings indicate that subjective bodily experience is an integral component of music-induced emotions, possibly contributing to the intensity of these emotions and providing distinction between different emotional states.

Canonical basic emotions are viewed as evolved reactions to situations relevant for survival (73), whereas music-induced emotions might not serve specific survival functions and could rely on different or only some of the mechanisms giving rise to more prototypical emotions (74). Pattern classification of fMRI data implies a partly distinct basis for music-induced and other emotions: Music-induced emotions are associated with relatively unspecific activation patterns outside the sensory and motor cortices (34), while emotions evoked by non-musical stimuli such as movies can be decoded from widely distributed activation patterns across many cortical and subcortical regions (75). In the current study, the topographies of music-induced bodily sensations appeared less discrete across emotion categories than those for biologically relevant emotional stimuli (35). The greatest overlap between BSMs was observed for the sad and tender songs. This was probably because they both induced a low-arousal positive emotional state in line with the well-known “paradox” of pleasurable sadness in music (76). Thus, even though listeners may label their affective responses to music as sadness, our results suggest these affective states are relatively undifferentiated in terms of bodily sensations from positive emotions.

## Limitations

The current study compared only two cultures. Although we deliberately drew subjects from two distant cultures (Western Europe and North America versus East Asia), these data do not capture all the possible culture-dependent variation in musical bodily feelings. While the two cultures were not completely isolated from each other, the East-Asian participants were markedly less familiar with the Western songs, which are widely known in the West, while the Western participants exhibited very low familiarity with the East-Asian songs. Our musical stimuli were mainly drawn from popular music and future studies could employ music from more diverse musical traditions (77). Future studies could quantify and systemically vary how closely the musical stimuli follow statistical regularities in different musical cultures and test how these cultural distances affect music-induced emotions and bodily sensations in listeners (78). Finally, our study was focused on self-reports. However, there is individual variation interoceptive ability and subjective bodily experience may not always accurately represent physiological changes in the body. Although the subjective experience was our primary interest here, future studies could link the self-reported bodily sensations with direct measurements of ANS activity using e.g., psychophysiological measures or total-body PET imaging (79).

## Conclusions

We conclude that music induces consistent bodily sensations and emotions across the studied Western and East Asian cultures. These subjective feelings were similarly associated with acoustic and structural features of music in both cultures. These results demonstrate similar embodiment of music-induced emotions in geographically distant cultures and suggest that music-induced emotions transcend cultural boundaries due to cross-culturally shared emotional connotations of specific musical cues. We argue that bodily experience, which may arise from skeletomuscular activity and changes in the physiological state of the body, plays a critical role in the elicitation and differentiation of music-induced emotions.

## Supplementary Material

Appendix 01 (PDF)

## Data Availability

Anonymized code and data have been deposited in OSF (DOI: 10.17605/OSF.IO/8BKPX) (80).
