# Supplementary material for: Bodily maps of musical sensations across cultures
Source: Proc Natl Acad Sci U S A. 2024 Jan 25;121(5):e2308859121. doi: 10.1073/pnas.2308859121 (PMC10835118; doi:10.1073/pnas.2308859121)
Supplement: Supplementary file 1 — Appendix 01 (PDF) [file pnas.2308859121.sapp.pdf]

PNAS

Supporting Information for
Bodily maps of musical sensations across cultures

Vesa Putkinen, Xinqi Zhou, Xianyang Gan, Linyu Yang, Benjamin Becker, Mikko Sams, Lauri Nummenmaa

Address Correspondence to
Vesa Putkinen
Email: vesa.putkinen@utu.fi
Benjamin Becker
Email: ben_becker@gmx.de

This PDF file includes:

Supporting text
Figures S1 to S7
Tables S1 to S2
SI References

Other supporting materials for this manuscript include the following:

Data and code are available at https://osf.io/8bkpx/

Supporting Information Text

Culture, music, and emotions

We investigated the relationship between the spatial patterns of music-induced bodily sensations and the emotional and acoustic attributes of music in a cross-cultural replication approach with participants from Western regions (United States and Western Europe) and East-Asian regions (China), using both Western and East Asian music. We refer to 'Western' and 'East-Asian' as cultures for heuristic purposes but acknowledge that these terms do not fully capture diverse musical backgrounds of the participants and that there exists a rich diversity of musical exposure within these cohorts, and that individuals within them may have encountered multiple musical cultures and possibly identify with various sub-cultures. We argue that, despite these caveats, the participants within each cohort are more likely to share not only geographical location, nationality, and language but also customs, norms and beliefs as well as aspects of musical exposure that may have relevance for music-induced emotions. In accordance with the recommendations by Jacoby et al. (1) local experts were enlisted for guidance in determining the cohorts to be studied and the stimulus materials. Furthermore, we explicitly define music and emotions below to elucidate the underlying assumptions of the study.

Music is a form of art and cultural expression that involves organized sounds created through the manipulation of various elements that may include pitch, rhythm, dynamics, timbre and lyrics. Music is often performed and appreciated for its aesthetic, emotional, and communicative qualities. There is cultural variation in the relative importance of specific musical elements, like harmony, and what qualifies as music.

Emotions are psychological and physiological states induced by internal or external events that involves subjective feelings, physiological, and behavioral responses. Emotions can range from putatively universal states experienced as happiness, sadness, anger, fear, and disgust to more complex and nuanced feelings. There is individual, cultural and contextual variation in the intensity, duration, physiological and neural underpinnings and expression of emotions. Music-induced emotions are often conceptualized as discrete categories (2) but these categories may have fuzzy boundaries (3) and/or may reflect more elemental dimensions like valence and arousal (4).

Supplementary Methods

Similarity analysis of the rating data

To conduct a similarity analysis of the dimensional ratings across the two cultures, we first calculated the mean ratings for each dimension separately for each song for both Western and East-Asian participants. Then we computed the mean values within each category (happy, sad, scary, tender, aggressive, and danceable) for both sets of stimuli (Western and Asian songs). Finally, we organized the resulting 120 category-specific mean ratings (6 categories × 2 stimulus sets × 10 dimensions) into arrays for both Western and East-Asian participants and determined the correlation between these arrays. These results are depicted in Figure 1 in the main text.

Comparisons of dimension-wise ratings between Western and East Asian participants

The continuous ratings were analyzed separately for each of the 11 dimensions with linear mixed model with Culture (Western, East Asian), Stimulus Set (Western vs. East Asian songs), Category (Aggressive, Danceable, Happy, Sad, Scary and Tender songs) as fixed factors and subject as the random factor. The analyses were performed using the lme4, lmerTest and emmeans packages in R. These results are reporter in the supplementary results below.

Similarity analyses across ratings and BSMs

We generated distance matrices (Euclidean distance) for the 144 song-wise BSMs (72 Western and 72 East Asian songs) and the corresponding dimensional ratings separately for the Western and East Asian participants. This yielded four 144 × 144 distance matrices: one BSM and one rating distance matrix for the Western and East Asian participants. The correlations between the matrices were computed using Mantel's test. The results are illustrated in Figure 3 of the main text.

Hierarchical Clustering

To further examine the similarity in the organization of the dimension ratings and BSMs across the tested cultures, we performed hierarchical clustering of the ratings and BSMs using the hclust function in R. Here were used the average ratings and BSMs per category (happy, sad, scary, tender, aggressive, and danceable) for both stimulus sets (Western and Asian songs). The clustering was performed separately for the ratings and BSMs and for the Western and East Asian subjects. These results are illustrated in Figure 4.

Correlation analysis for ratings and BSMs

We first calculated the mean BSMs for each of the 72 songs (song-wise BSMs) and mean ratings for each song (song-wise ratings) for each of the 10 dimensions. The song-wise ratings were then subjected to principal component analysis (PCA). Two principal components explained over 90% of the variance in both cultures and were included in the subsequent analyses. Then, for every pixel, we computed a correlation involving 72 data points representing the BSM "activation" in that particular pixel for each song and another set of 72 data points representing the scores for one of the PCs for each song (pixel-wise correlations). This procedure was repeated for each pixel within the body silhouette (N = 62320) and for both PCs. False Discovery Rate (FDR) was used to control for false positives. These results are shown in Figure 5.

Similarity analysis for musical features

We computed mean ratings per each song and dimension and extracted features listed in supplementary table S2 for each song using the MIR toolbox. In total, there were 144 songs, and each song had a mean rating for each of the 10 dimension (shown in the X-axis in Figure 6) and a single value for each of 21 music features (listed on the Y-axis of Figure 6). First, we computed the correlation for every dimension-feature pair. We then arranged these correlations as an array both for the Western and East-Asian participants and computed the correlation between the arrays. These results are shown in Figure 6 of the main text.

Correlation analysis for musical features and BSMs

We first calculated the mean BSMs and extracted the musical features listed in supplementary Table S2 for each song. The song-wise feature values were then subjected to principal component analysis. Three components were explained 58% of the variance and were included in the subsequent analyses. For every pixel, we computed a correlation involving 72 data points representing the BSM "activation" in that particular pixel for each song and another set of 72 data points representing the scores for one of the PCs for each song. This procedure was repeated for each pixel within the body silhouette (N = 62320) and for the three PCs. FDR was used to control for false positives. These results are shown in Figure 7.

Comparisons of dimension-wise ratings between Western and East Asian participants

The tables below list the results of LMM analyses for each dimension. The figures display the estimated means and 95% confidence intervals for each dimension per song category, culture (circle = Asian subjects, triangle = Western subjects) and stimulus set (blue = Asian songs, red = Western songs).

Despite the high correlation between the emotion ratings between Western and East-Asian participants (**Figure 1 and Figure 2**), the dimension-wise LMM analysis revealed some differences between the tested cultures. Most notable ones were as follows: The East Asian subjects rated the happy, danceable and tender songs higher for tenderness than the Western participants; The Western participants gave lower ratings for fear for the scary songs in general and East Asian scary songs in particular than the East-Asian participants; Similarly, the Western subject gave lower ratings for sadness particularly for the East Asian sad songs. For happiness, liking, danceability, relaxation and energization, the Western participants gave lower ratings for the East-Asian happy and danceable songs. Conversely, the Western subjects rated these songs higher for irritation than the East-Asian participants did. The East Asian subjects in turn, rated the scary songs lower on liking and higher on irritation and aggression than the Western subjects. The East-Asian subjects rated the tender songs higher in energization than the Western subjects did. Finally, as mentioned in the main text, Western subjects were more familiar with the Western songs while East Asian subject were more familiar with the Asian songs.

Tenderness

	Sum Sq	Mean Sq	NumDF	DenDF	F value	Pr(>F)
Stimulus Set	7.878267e+01	78.78267	1	10499.5970	0.1904993	0.6625104
Culture	4.756614e+04	47566.13512	1	831.3289	115.0166019	0.00000
Song Category	4.004954e+06	800990.80103	5	10499.5970	1936.8241682	0.00000
Stimulus Set*Culture	2.686700e+03	2686.70007	1	10499.5970	6.4965361	0.0108226
Stimulus Set*Song Category	1.401799e+05	28035.97194	5	10499.5970	67.7919746	0.00000
Culture*Song Category	1.984895e+05	39697.90178	5	10499.5970	95.9909346	0.00000
Stimulus Set*Culture*Song Category	1.876344e+03	375.26883	5	10499.5970	0.9074134	0.4749836

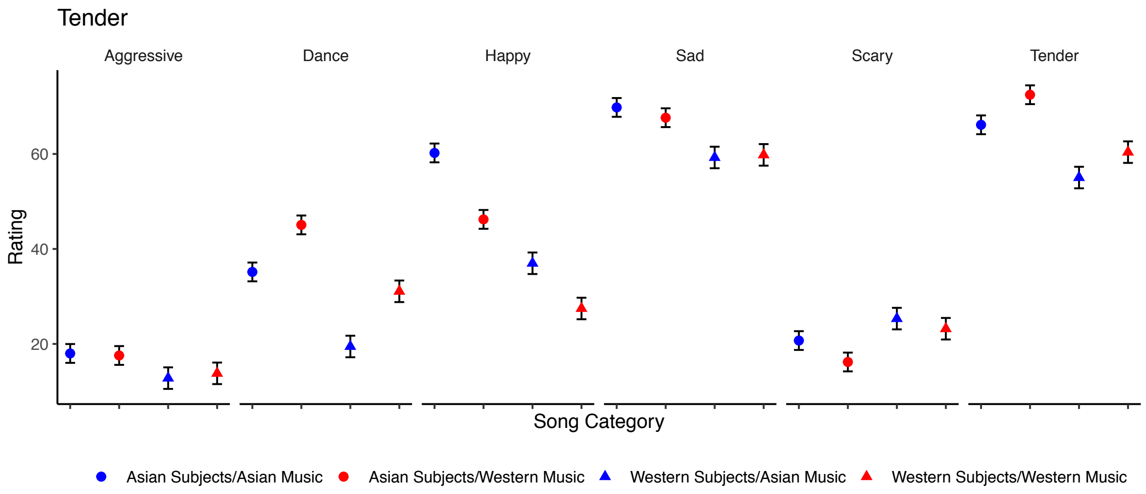

Fear

	Sum Sq	Mean Sq	NumDF	DenDF	F value	Pr(>F)
Stimulus Set	12813.351	12813.351	1	10488.5288	36.755285	0.00000
Culture	6470.229	6470.229	1	823.4615	18.559945	0.0000185
Song Category	5090435.923	1018087.185	5	10488.5288	2920.397861	0.00000
Stimulus Set*Culture	1562.499	1562.499	1	10488.5288	4.482052	0.0342760
Stimulus Set*Song Category	24019.087	4803.817	5	10488.5288	13.779820	0.00000
Culture*Song Category	120202.110	24040.422	5	10488.5288	68.960300	0.00000
Stimulus Set*Culture*Song Category	11202.584	2240.517	5	10488.5288	6.426955	0.0000057

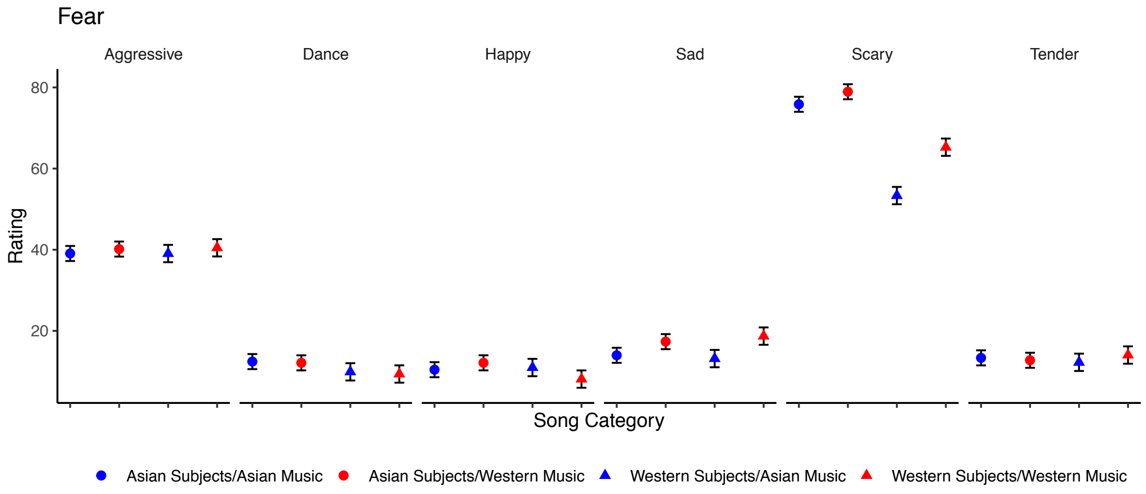

Sadness

	Sum Sq	Mean Sq	NumDF	DenDF	F value	Pr(>F)
Stimulus Set	9.338506e+01	93.38506	1	10489.7753	0.207064	0.6490878
Culture	6.399550e+03	6399.54987	1	818.6953	14.189814	0.0001771
Song Category	3.050642e+06	610128.34500	5	10489.7753	1352.846381	0.00000
Stimulus Set*Culture	1.279465e+04	12794.65017	1	10489.7753	28.369762	0.0000001

Stimulus Set*Song Category	3.550696e+04	7101.39174	5	10489.7753	15.746018	0.00000
Culture*Song Category	3.651380e+04	7302.76046	5	10489.7753	16.192516	0.00000
Stimulus Set*Culture*Song Category	7.183615e+04	14367.22932	5	10489.7753	31.856665	0.00000

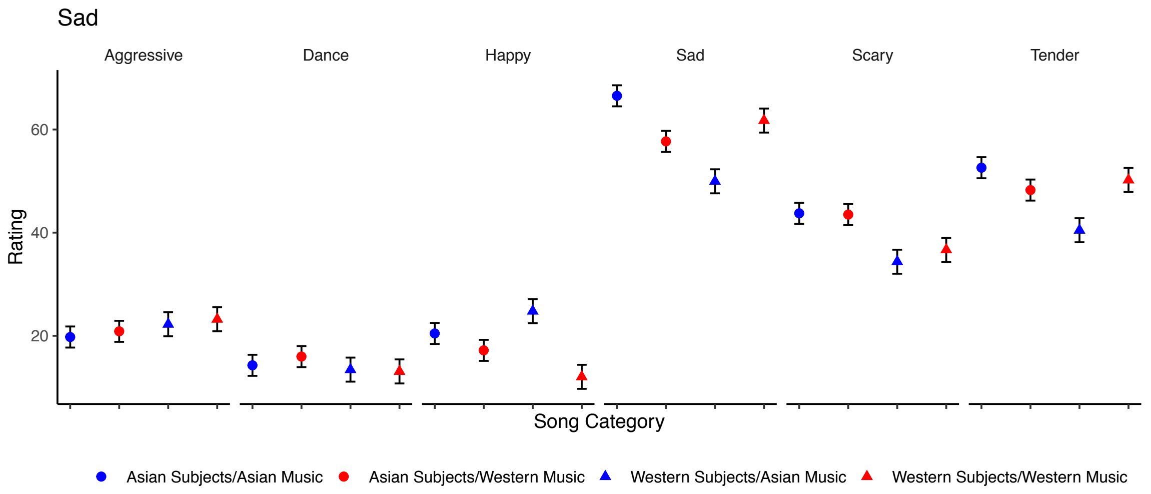

Happiness

	Sum Sq	Mean Sq	NumDF	DenDF	F value	Pr(>F)
Stimulus Set	33593.095	33593.095	1	10487.408	74.81021	0.00000
Culture	4729.069	4729.069	1	815.137	10.53141	0.0012215
Song Category	3735192.480	747038.496	5	10487.408	1663.61891	0.00000
Stimulus Set*Culture	9537.875	9537.875	1	10487.408	21.24039	0.0000041
Stimulus Set*Song Category	52096.673	10419.335	5	10487.408	23.20336	0.00000
Culture*Song Category	60443.798	12088.760	5	10487.408	26.92109	0.00000
Stimulus Set*Culture*Song Category	58750.235	11750.047	5	10487.408	26.16679	0.00000

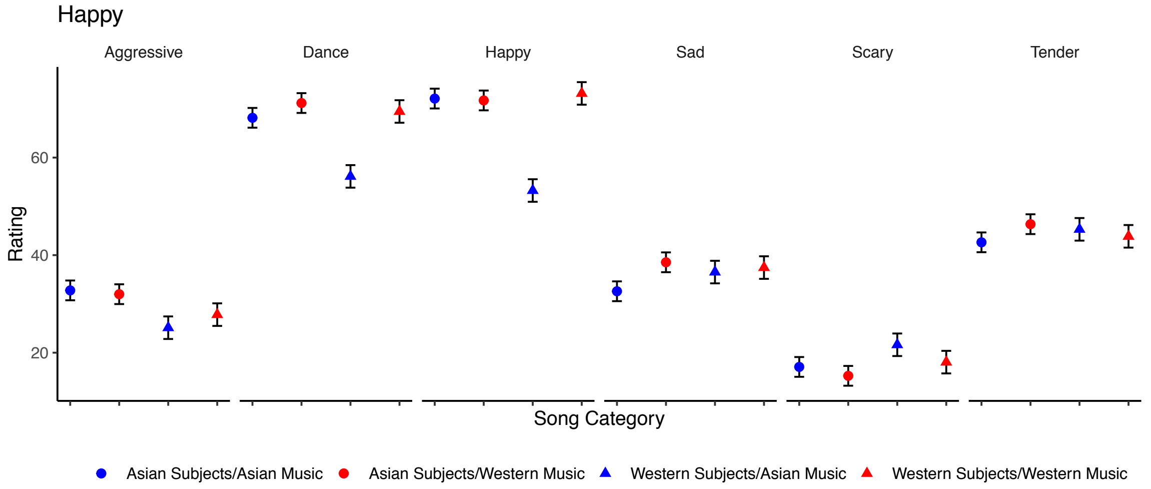

Danceability

	Sum Sq	Mean Sq	NumDF	DenDF	F value	Pr(>F)

Stimulus Set	44176.329	44176.329	1	10491.4264	86.78469	0.00e+00
Culture	31058.726	31058.726	1	819.0918	61.01508	0.00e+00
Song Category	4548642.585	909728.517	5	10491.4264	1787.16777	0.00e+00
Stimulus Set*Culture	8778.939	8778.939	1	10491.4264	17.24628	3.31e-05
Stimulus Set*Song Category	132613.229	26522.646	5	10491.4264	52.10392	0.00e+00
Culture*Song Category	28716.234	5743.247	5	10491.4264	11.28265	0.00e+00
Stimulus Set*Culture*Song Category	49650.292	9930.058	5	10491.4264	19.50767	0.00e+00

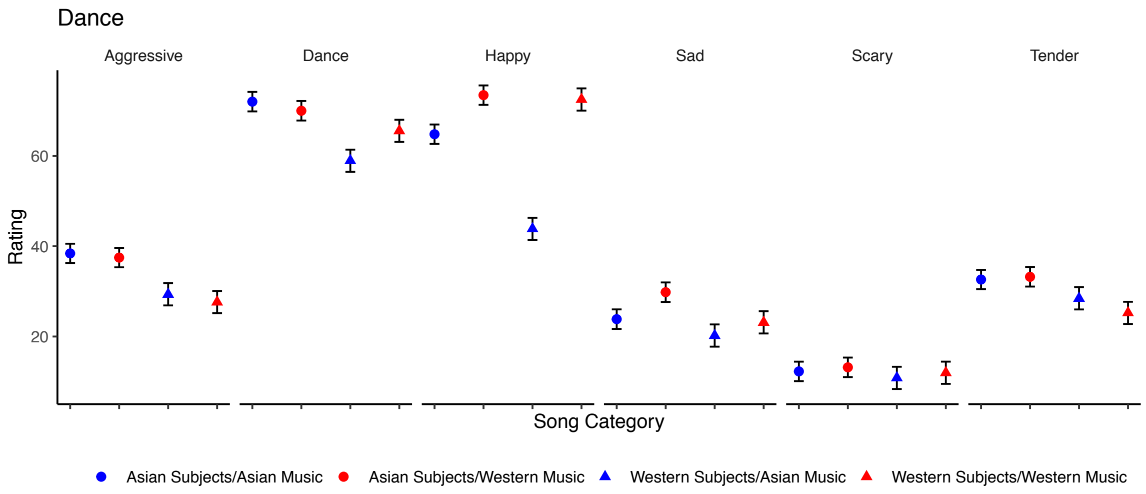

Aggressiveness

	Sum Sq	Mean Sq	NumDF	DenDF	F value	Pr(>F)
Stimulus Set	1.413393e+04	1.413393e+04	1	10474.2078	37.761499	0.00000
Culture	1.992588e+04	1.992588e+04	1	804.0993	53.235810	0.00000
Song Category	7.218014e+06	1.443603e+06	5	10474.2078	3856.860784	0.00000
Stimulus Set*Culture	9.383718e+01	9.383718e+01	1	10474.2078	0.250704	0.6165903
Stimulus Set*Song Category	8.706929e+04	1.741386e+04	5	10474.2078	46.524454	0.00000
Culture*Song Category	3.348005e+05	6.696011e+04	5	10474.2078	178.896735	0.00000
Stimulus Set*Culture*Song Category	4.083564e+03	8.167129e+02	5	10474.2078	2.182005	0.0532778

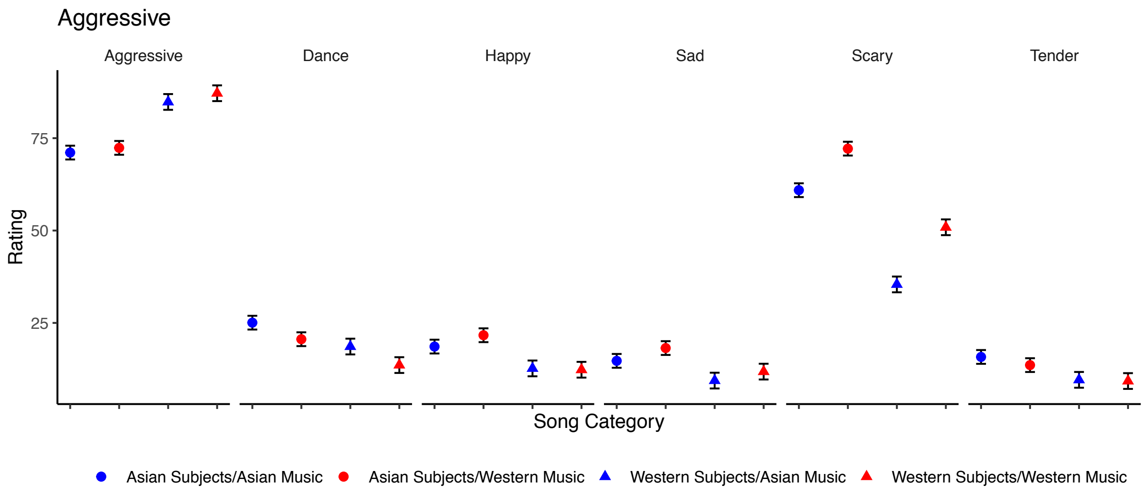

Liking

	Sum Sq	Mean Sq	NumDF	DenDF	F value	Pr(>F)
Stimulus Set	112883.809	112883.809	1	10500.1944	198.467302	0.000000
Culture	4004.445	4004.445	1	815.6394	7.040437	0.008124
Song Category	3751696.728	750339.346	5	10500.1945	1319.213329	0.000000
Stimulus Set*Culture	79961.153	79961.153	1	10500.1944	140.584150	0.000000
Stimulus Set*Song Category	79730.901	15946.180	5	10500.1945	28.035866	0.000000
Culture*Song Category	138482.685	27696.537	5	10500.1945	48.694822	0.000000
Stimulus Set*Culture*Song Category	50351.012	10070.202	5	10500.1945	17.704983	0.000000

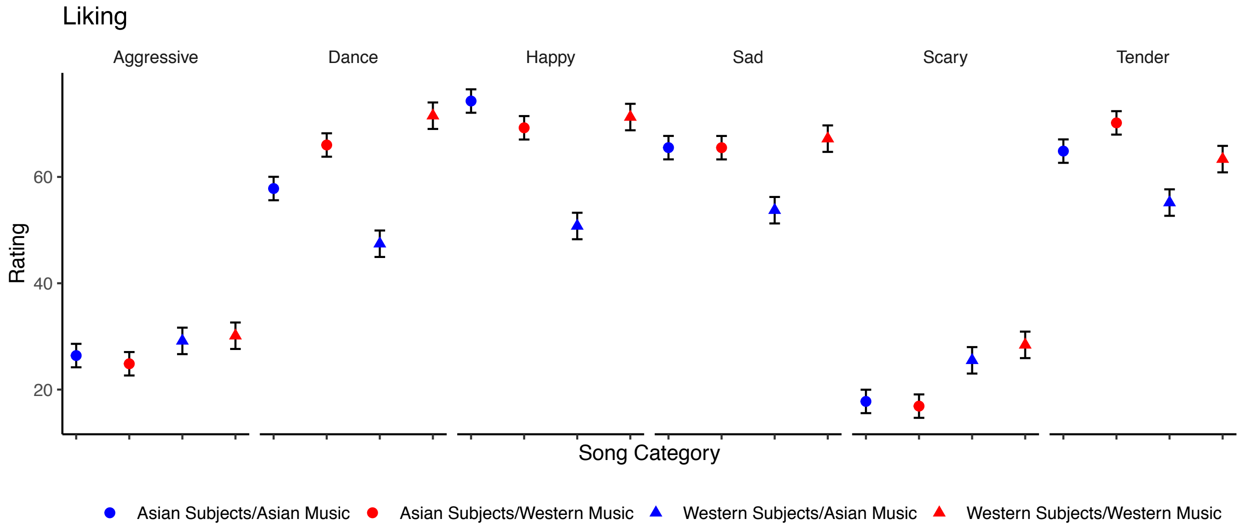

Irritation

	Sum Sq	Mean Sq	NumDF	DenDF	F value	Pr(>F)
Stimulus Set	64077.467	64077.467	1	10509.52	105.853222	0.00e+00
Culture	9795.574	9795.574	1	831.92	16.181867	6.28e-05
Song Category	3267626.840	653525.368	5	10509.52	1079.595834	0.00e+00

Stimulus Set*Culture	42380.634	42380.634	1	10509.52	70.010987	0.00e+00
Stimulus Set*Song Category	81795.137	16359.027	5	10509.52	27.024410	0.00e+00
Culture*Song Category	212054.866	42410.973	5	10509.52	70.061106	0.00e+00
Stimulus Set*Culture*Song Category	16768.166	3353.633	5	10509.52	5.540058	4.23e-05

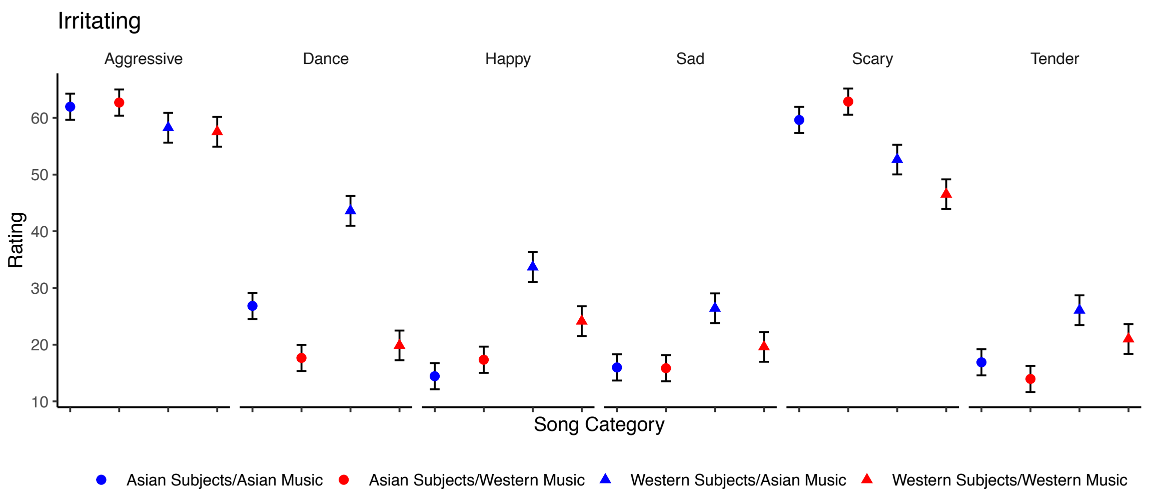

Energizing

	Sum Sq	Mean Sq	NumDF	DenDF	F value	Pr(>F)
Stimulus Set	55618.542	55618.542	1	10488.5003	117.97472	0.00e+00
Culture	34452.877	34452.877	1	814.2813	73.07938	0.00e+00
Song Category	3834177.172	766835.434	5	10488.5003	1626.56535	0.00e+00
Stimulus Set*Culture	8171.128	8171.128	1	10488.5003	17.33211	3.16e-05
Stimulus Set*Song Category	117138.733	23427.747	5	10488.5003	49.69353	0.00e+00
Culture*Song Category	137747.393	27549.479	5	10488.5003	58.43630	0.00e+00
Stimulus Set*Culture*Song Category	42841.295	8568.259	5	10488.5003	18.17448	0.00e+00

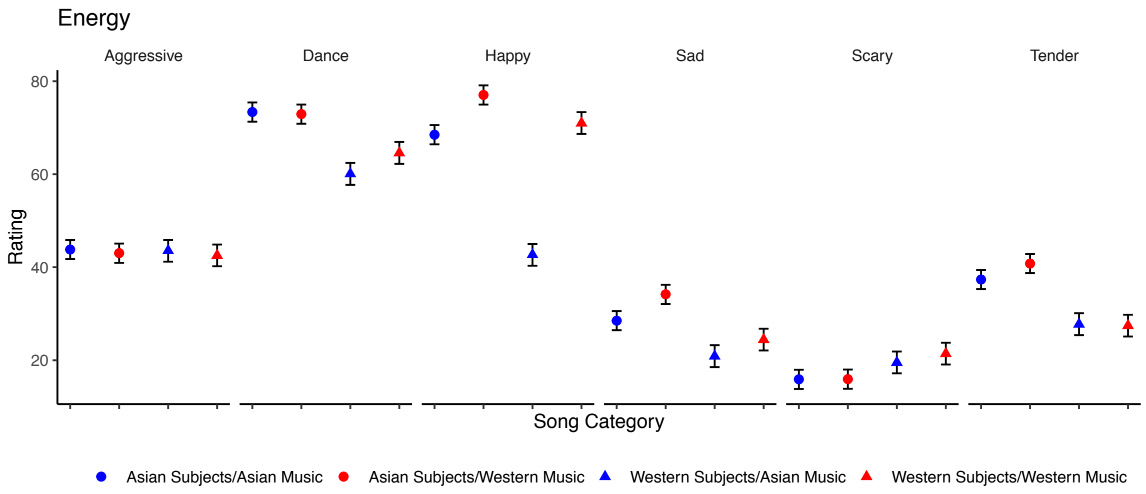

Relaxing

	Sum Sq	Mean Sq	NumDF	DenDF	F value	Pr(>F)
Stimulus Set	20757.080	20757.080	1	10497.7476	44.803966	0.00000
Culture	86345.245	86345.245	1	820.9061	186.375415	0.00000
Song Category	3938836.789	787767.358	5	10497.7476	1700.388568	0.00000
Stimulus Set*Culture	3293.436	3293.436	1	10497.7476	7.108852	0.0076822
Stimulus Set*Song Category	114416.863	22883.373	5	10497.7476	49.393548	0.00000
Culture*Song Category	276177.311	55235.462	5	10497.7476	119.225235	0.00000
Stimulus Set*Culture*Song Category	48315.755	9663.151	5	10497.7476	20.857822	0.00000

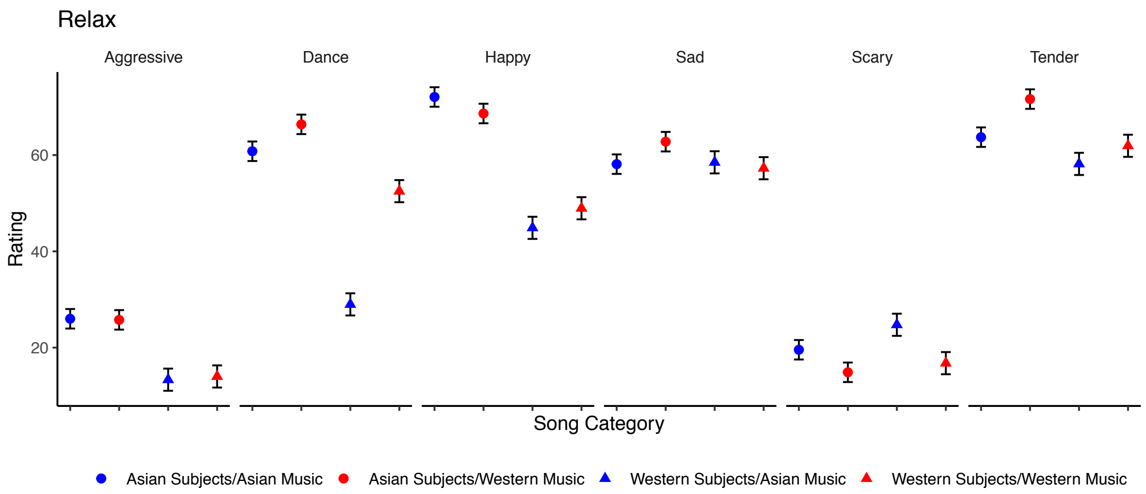

Familiar

	Sum Sq	Mean Sq	NumDF	DenDF	F value	Pr(>F)
Stimulus Set	110423.82	110423.819	1	10476.7352	159.52902	0
Culture	39833.21	39833.213	1	801.9935	57.54695	0
Song Category	2373461.93	474692.386	5	10476.7352	685.78693	0
Stimulus Set*Culture	1367466.09	1367466.087	1	10476.7352	1975.57492	0

Stimulus Set*Song Category	44208.38	8841.676	5	10476.7352	12.77355	0
Culture*Song Category	146187.37	29237.473	5	10476.7352	42.23931	0
Stimulus Set*Culture*Song Category	778035.04	155607.007	5	10476.7352	224.80506	0

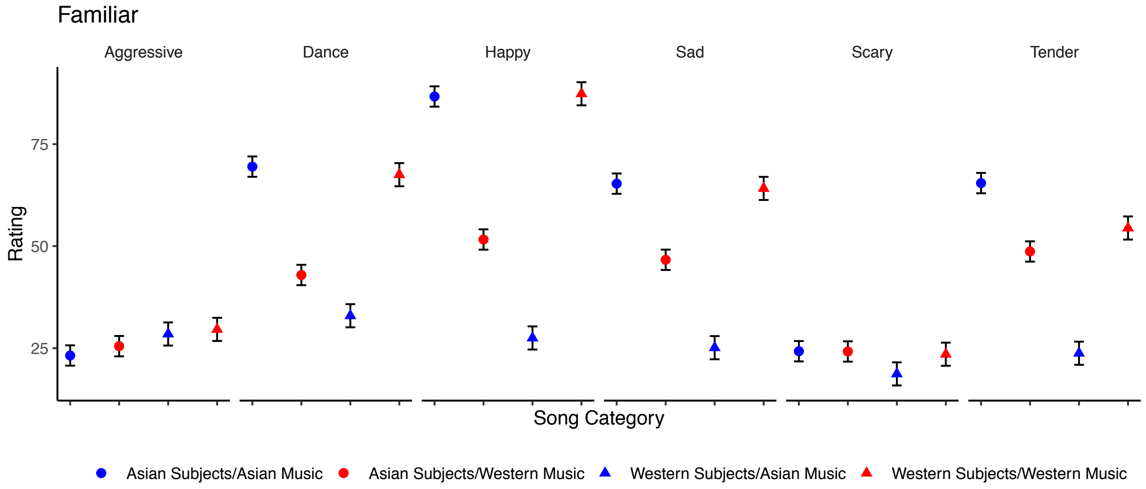

Supplementary discussion

An often-adopted distinction in cross-cultural research involves categorizing cultures as either "individualist" or "collectivist," depending on whether they prioritize individual needs or in-group goals. Western cultures, such as the United States are typically classified as individualist whereas Asian cultures, such as China, as collectivist (5). As in our data, one study found that participants from ‘collectivist’ countries (Brazil, Kenya, and Portugal) reported higher prevalence of music-induced positive emotions (happiness) than those from ‘individualist’ Western countries (Australia, Sweden, and USA) while the reverse was true for nominally negative emotions (sadness) (5, see also ref 6). Speculatively, higher prevalence of experiencing positive vs. negative emotions in response to music could give rise to more differentiated bodily responses such emotions as exemplified here by the stronger bodily responses in the Western versus East Asian participants for the sad and aggressive songs. Accordingly, the Western subjects gave higher ratings than the East-Asian participants predominantly for the nominally negative categories (sad, fear, aggressive) and emotions (e.g., irritation). There are numerous other possible sources for cultural differences in emotional and bodily responses to music such as cultural variation in how different musical activities (e.g., dance) or genres (e.g., aggressive metal or punk) are valued, more general cultural attitudes towards expressing certain emotions or even linguistic metaphors used to describe emotions (“Butterflies in the stomach", “gut-wrenching”) to name a few. The numerous potential factors driving the observed cultural differences render the interpretation of these differences speculative, but also emphasize the robustness of the observed cultural similarities.

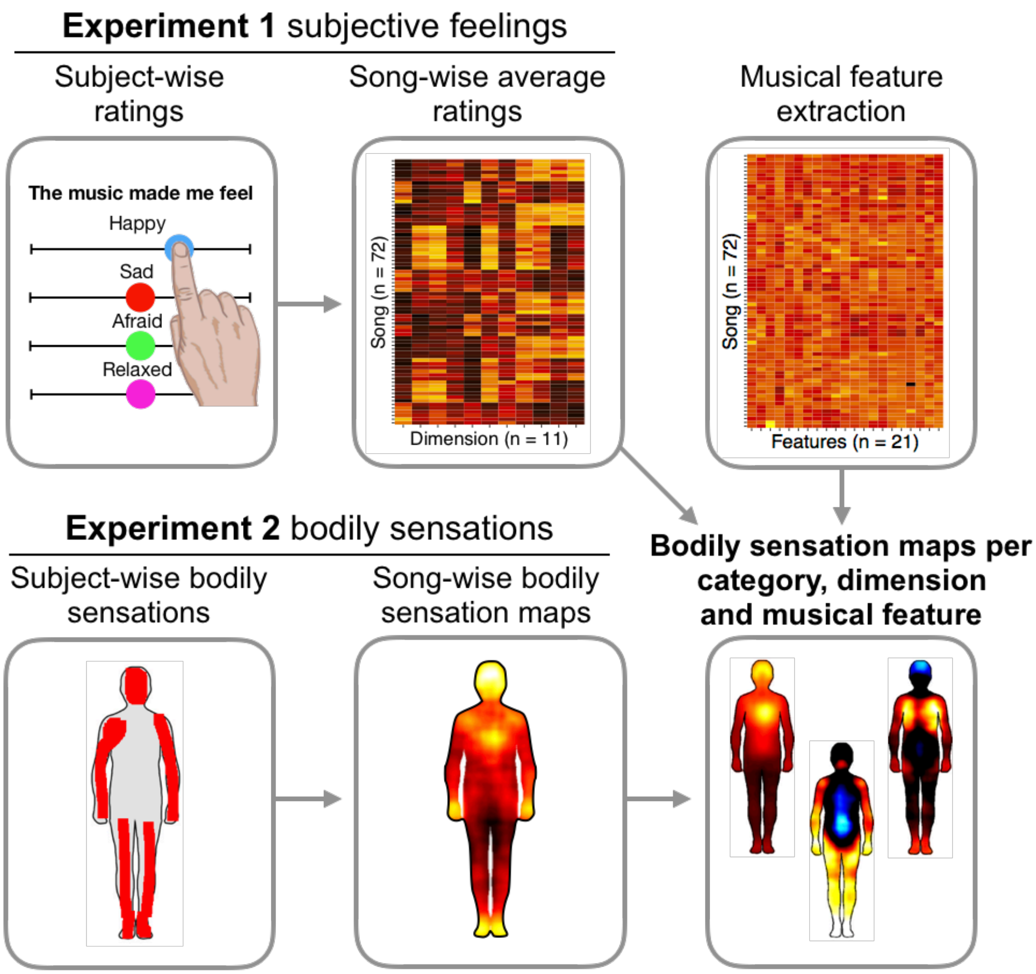

Fig. S1. Analysis workflow

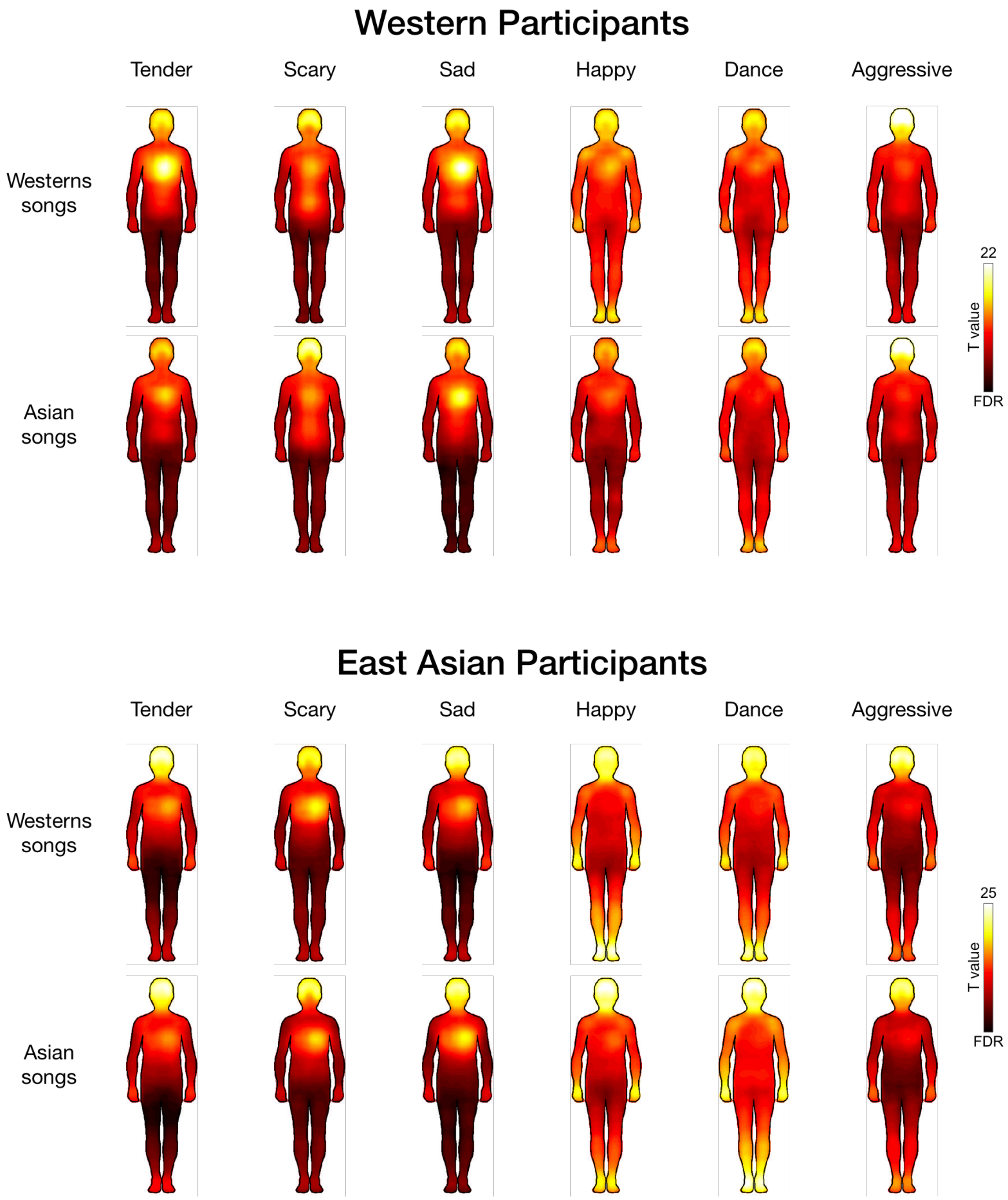

Fig. S2. BSMs for Western and Asian songs in Western and East Asian participants.

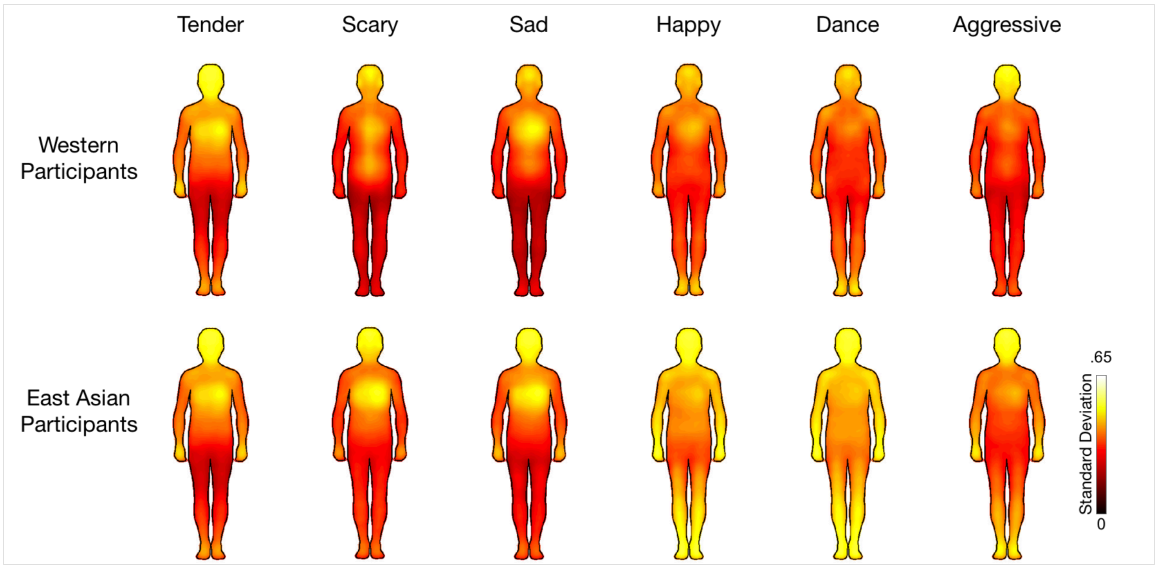

Fig. S3. Standard deviation maps for Western and East Asian participants

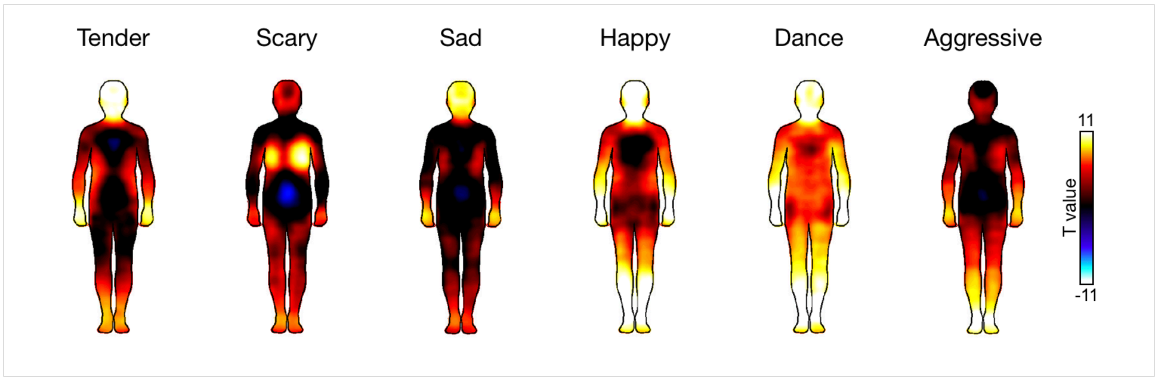

Fig. S4. East Asian-minus-Western difference maps.

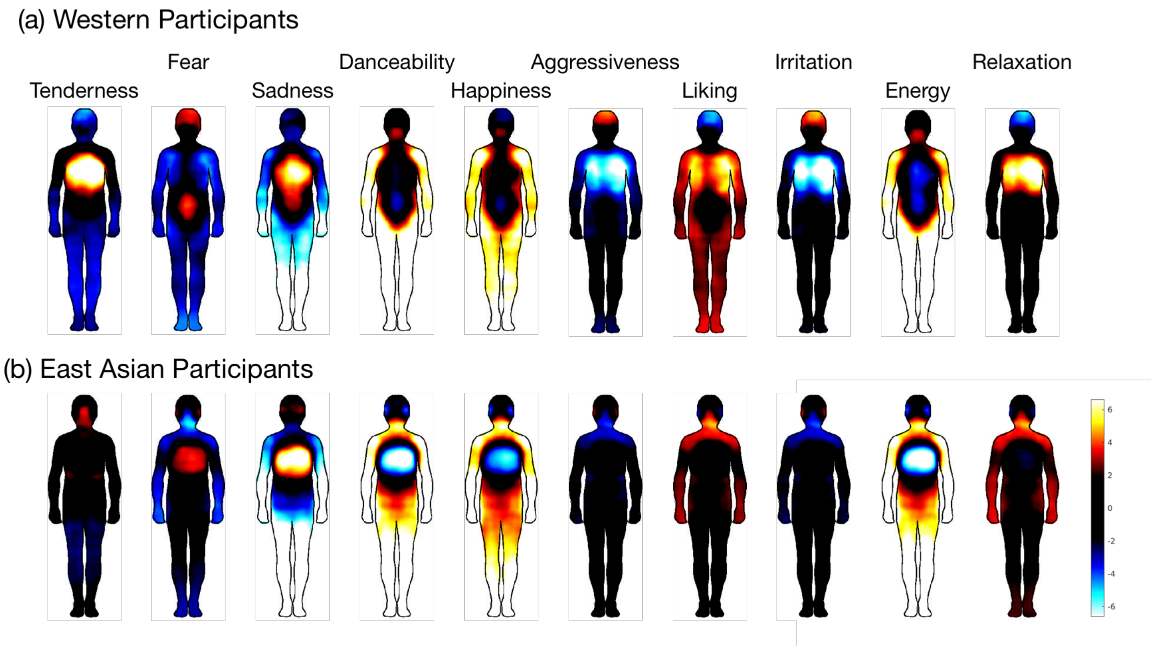

Fig. S5. BSMs for the 10 continuous dimensions in Western (a) and East Asian participants (b). Colourbar indicates T-value.

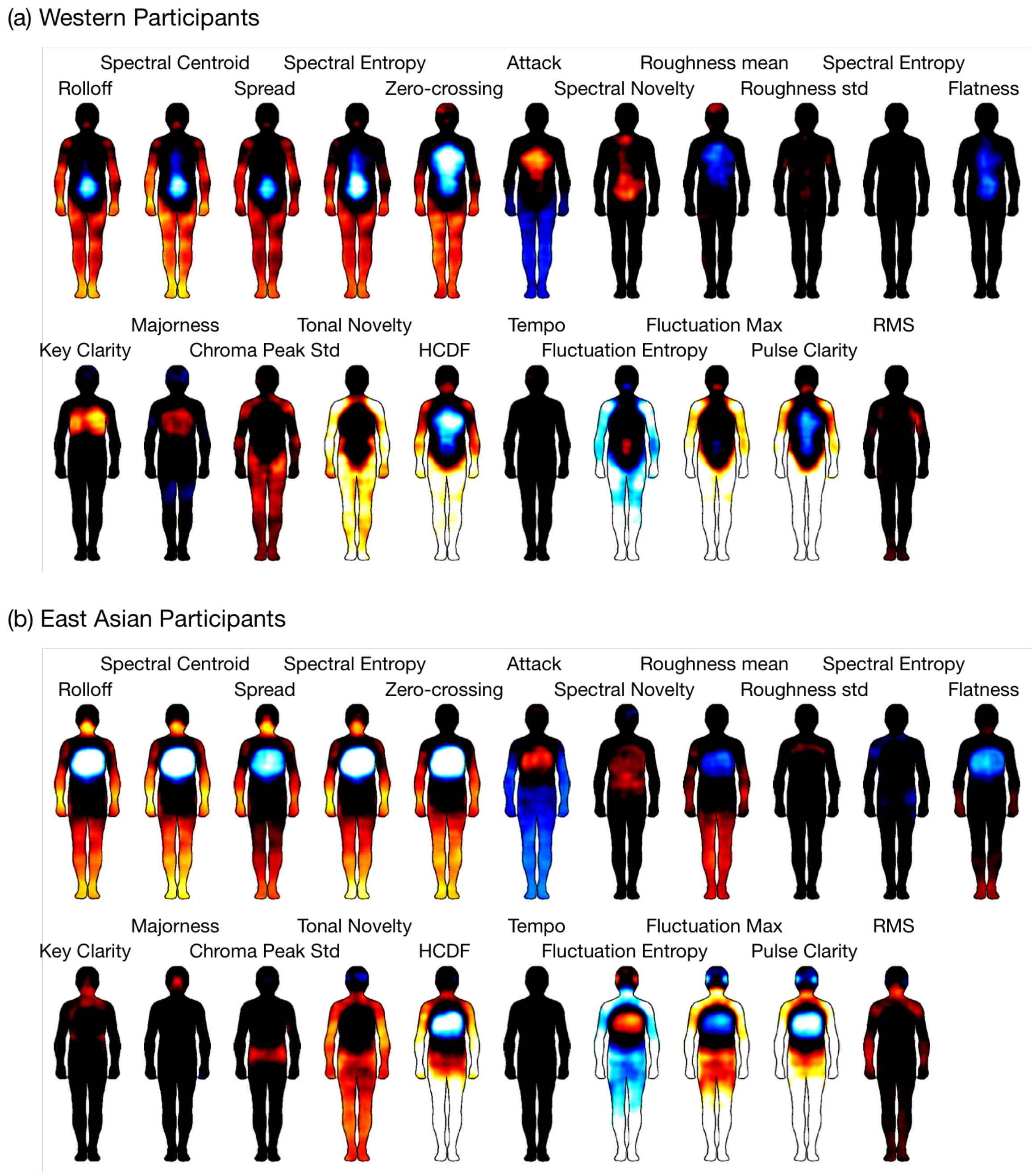

Fig. S6. Correlation BSMs for the individual musical features.

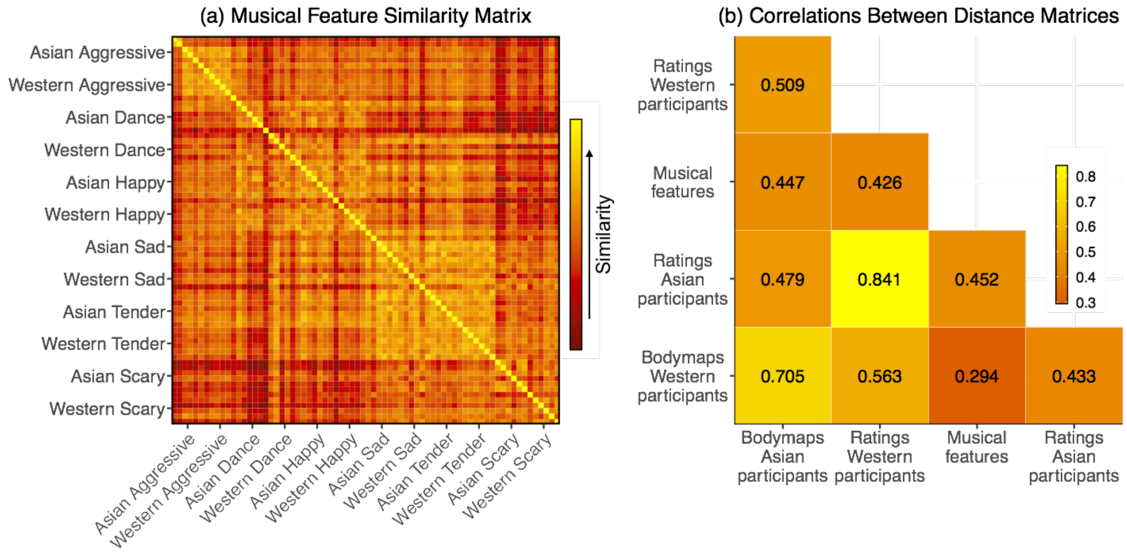

Fig. S7. a) Song-by-song musical feature similarity matrix. (b) correlations between BSMs, rating and musical feature similarity matrices.

Table S1. Musical pieces used as stimuli.

	Western songs			
Category	Artist	Title	Timing	Lyrics
Aggressive	Carnifex	Lie to My Face	0:00-0:26	Yes
Aggressive	Dying Fetus	Panic Amongst the Herd	1:13-1:38	Yes
Aggressive	High on Fire	Spewn from the Earth	0:25-0:52	Yes
Aggressive	Meshuggah	Bleed	5:52-6:27	Yes
Aggressive	Pantera	Strength Beyond Strength	1:10-1:43	Yes
Aggressive	Slayer	Angel of Death	1:04-1:39	Yes
Dance	Rachel Portman	Oliver Learns the Hard Way	1:39-2:09	No
Dance	Jean-Yves Thibaude	Meryton Townhall	0:33-1:06	No
Dance	Jubël	Dancing in the Moonlight	0:56-1:28	Yes
Dance	LaBelle	Lady Marmalade	0:16-0:48	Yes
Dance	Parliament	Flash Light	0:04-0:38	Yes
Dance	Stevie Wonder	Superstition	0:28-1:20	Yes
Happy	ABBA	Mamma Mia	0:15-0:49	Yes
Happy	Bryan Adams	Summer of '69	1:10-1:40	Yes
Happy	Bruno Mars	The Lazy Song	0:49-1:21	Yes
Happy	Pharrell Williams	Happy	2:00-2:36	Yes
Happy	Taylor Swift	Shake It Off	0:00-0:28	Yes
Happy	Zara Larsson	Lush Life	1:46-2:26	Yes
Sad	Adele	Someone Like You	0:12-0:45	Yes
Sad	Academy of St: Martin in the Fields	Ask your Saint Who He's Killed	0:00-0:38	No
Sad	Christina Perri	Jar of Hearts	0:00-0:38	Yes
Sad	Rihanna	Stay	0:51-1:34	Yes
Sad	Benjamin Wallfisch	Darcy's Letter	0:40-1:12	No
Sad	Lady Gaga & Bradley Cooper	Shallow	1:04-1:45	Yes
Scary	Danny Elfman	Selina Transforms Pt: I	0:10-0:46	No
Scary	Steve Jablonsky	Freddy's Coming for You	1:01-1:37	No

Scary	Steve Jablonsky	Freddy's Coming for You	2:18-2:54	Yes
Scary	György Ligeti	Requiem For Soprano, Mezzo-Soprano, Two Mixed Choirs & Orchestra	2:13-2:43	Yes
Scary	Jerry Goldsmith	The Killer Storm	0:49-1:20	Yes
Scary	Howard Shore	Whipping	1:34-2:04	No
Tender	Jeff Buckley	Hallelujah	0:59-1:26	Yes
Tender	William Coulter	Citi Na GCumman	3:21-3:46	No
Tender	CUTTS	Breathe	2:23-3:01	Yes
Tender	Adrian Legg	Hymn for Jaco	1:35-2:15	No
Tender	Tom Spleight	Save Tonight	0:06-0:38	Yes
Tender	Wiz Khalifa	See You Again	0:00-0:38	Yes

East Asian Songs

Category	Artist	Title	Timing	Lyrics
Aggressive	郁乐队 (yu yue dui)	在风暴的中央 (zai feng bao de zhong yang)	0:53-1:30	Yes
Aggressive	郁乐队 (yu yue dui)	异海之王 (yi hai zhi wang)	0:30-0:55	Yes
Aggressive	霾晦乐队 (mai hui yue dui)	禁忌之城 (jin ji zhi cheng)	1:01-1:35	Yes
Aggressive	霾晦乐队 (mai hui yue dui)	镜中世界 (jing zhong shi jie)	1:01-1:30	Yes
Aggressive	暗狱戮尸 (an yu lu shi)	成熟肉体的较量 (cheng shu rou ti de jiao liang)	0:11-0:41	Yes
Aggressive	Ephemerality-朝生暮死乐队 (Ephemerality- zhao sheng mu si yue dui)	人间弃子 (ren jian qi zi)	0:52-1:23	Yes
Dance	汪涵，马可 (wang han, ma ke)	多彩中国话 (duo cai zhong guo hua)	0:07-0:43	Yes

Dance	大张伟 (da zhang wei)	倍儿爽 (bei er shuang)	0:40-1:09	Yes
Dance	汪佩蓉 (wang pei rong)	头发湿的 (tou fa shi de)	2:23-3:09	Yes
Dance	郑秀文 (zheng xiu wen)	眉飞色舞 (mei fei se wu)	2:06-2:35	Yes
Dance	陈慧琳 (chen hui lin)	不如跳舞 (bu ru tiao wu)	0:46-1:14	Yes
Dance	花儿乐队 (hua er yue dui)	穷开心 (qiong kai xin)	0:52-1:31	Yes
Happy	陶喆 (tao zhe)	爱，很简单 (ai, hen jian dan)	1:08-1:37	Yes
Happy	陶喆，蔡依林 (tao zhe, cai yi lin)	今天你要嫁给我 (jin tian ni yao jia gei wo)	0:22-1:03	Yes
Happy	蔡依林 (cai yi lin)	日不落 (ri bu luo)	0:19-0:55	Yes
Happy	陈粒 (chen li)	易燃易爆炸 (yi ran yi bao zha)	1:39-2:22	Yes
Happy	汪苏泷，BY2 (wang su long, BY2)	有点甜 (you dian tian)	0:53-1:30	Yes
Happy	周杰伦 (zhou jie lun)	牛仔很忙 (niu zi hen mang)	0:15-0:46	Yes
Sad	林俊杰 (lin jun jie)	她说 (ta shuo)	0:24-1:11	Yes
Sad	丁当 (ding dang)	洋葱 (yang cong)	0:34-1:05	Yes
Sad	G:E:M:邓紫棋 (G:E:M: deng zi qi)	我要我们在一起 (wo yao wo men zai yi qi)	1:25-2:02	Yes
Sad	A-Lin (A-Lin)	有一种悲伤 (you yi zhong bei shang)	1:02-1:37	Yes

Sad	陈奕迅<br>(chen yi xun)	我们<br>(wo men)	0:48-1:20	Yes
Sad	魏如萱<br>(wei ru xuan)	窃笑<br>(qie xiao)	1:30-2:06	Yes
Scary	王德兵<br>(wang de bing)	鬼婴<br>(gui ying)	0:18-0:49	No
Scary	王德兵<br>(wang de bing)	恐怖鬼故事<br>(kong bu gui gu shi)	0:59-1:31	No
Scary	王德兵<br>(wang de bing)	恐怖鬼魂<br>(kong bu gui hun)	0:27-0:59	No
Scary	群星<br>(qun xing)	冥想<br>(ming xiang)	0:01-0:34	Yes
Scary	王德兵<br>(wang de bing)	三更夜<br>(san geng ye)	1:11-1:51	No
Scary	王德兵<br>(wang de bing)	死寂<br>(si ji)	3:02-3:37	No
Tender	张学友<br>(zhang xue you)	她来听我的演唱会<br>(ta lai ting wo de yan chang hui)	0:23-0:53	Yes
Tender	宋冬野<br>(song dong ye)	董小姐<br>(dong xiao jie)	0:14-0:49	Yes
Tender	李健<br>(li jian)	贝加尔湖畔<br>(bei jia er hu pan)	1:18-2:10	Yes
Tender	王若琳<br>(wang ruo lin)	一种念头<br>(yi zhong nian tou)	0:21-1:01	Yes
Tender	王铮亮<br>(wang zheng liang)	只愿你好<br>(zhi yuan ni hao)	0:59-1:29	Yes
Tender	薛之谦<br>(xue zhi qian)	丑八怪<br>(chou ba guai)	0:36-1:27	Yes

Table S2. Description of the musical features

Timbral features	
Attack	Attack duration averaged across the events detected in the amplitude envelope.
Spectral novelty	Estimate of the frequency and amount of change in the spectrum per time unit.
Roughness, standard deviation	The standard deviation across the stimulus of sensory dissonance estimated from the frequency ratios of each pair of peaks in the frequency spectrum.
Roughness, mean	Averaged sensory dissonance across the segment.
Spectral entropy	An index of the complexity of the spectrum.
Spectral entropy SC	An index of the complexity of the spectrum (estimated from a smoothed and collapsed spectrum).
Spectral roll-off	Frequency boundary below which 85% of the total energy is contained.
Spectral centroid	Geometric center on the frequency spectrum, i.e., the dominant frequency.
Spectral spread	Standard deviation of the frequency spectrum.
Spectral flatness	The ratio between the geometric mean and the arithmetic mean of the spectrum. An index of spikiness vs. flatness of the spectrum.
Zero crossing rate	The number of times the signal changes sign within a given time frame.
**Tonal Features**	
Key clarity	A measure of how clearly the pitch distribution of the song implies a specific key among all major and minor keys.
Majorness	Estimate of the modality (major vs. minor) of the song based on pitch distribution of the signal.
Chroma Peak Std	Standard deviation across the stimulus of the position of the maximum peak in the chromagram.
Tonal novelty	An estimate of frequency and amount of change in tonality per time unit.

HCDF	An index of harmonic change (variation in tonal centroid) in the signal (8).
**Rhythmic features**	
Tempo	Tempo estimated from periodicities in the amplitude envelope of the signal.
Fluctuation entropy	Shannon entropy of the fluctuation spectrum (i.e., periodicities at different frequency bands). High Fluctuation entropy indicates rhythmic complexity.
Pulse clarity	Rhythmic clarity estimated from the periodicities of the amplitude envelope of the signal (9).
Fluctuation max	Maximum of the summarized fluctuation spectrum.
**Dynamics**	
RMS	Variance of the root mean square (RMS) energy across the stimulus.

SI References

1. N. Jacoby, *et al.*, Cross-Cultural Work in Music Cognition. *Music Percept.* **37**, 185–195 (2020).

2. M. Zentner, D. Grandjean, K. R. Scherer, Emotions evoked by the sound of music: Characterization, classification, and measurement. *Emotion* **8**, 494–521 (2008).

3. A. S. Cowen, X. Fang, D. Sauter, D. Keltner, What music makes us feel: At least 13 dimensions organize subjective experiences associated with music across different cultures. *Proc. Natl. Acad. Sci.* **117**, 1924–1934 (2020).

4. T. Eerola, J. K. Vuoskoski, A comparison of the discrete and dimensional models of emotion in music. *Psychol. Music* **39**, 18–49 (2011).

5. M. B. Brewer, Y.-R. Chen, Where (who) are collectives in collectivism? Toward conceptual clarification of individualism and collectivism. *Psychol. Rev.* **114**, 133 (2007).

6. P. N. Juslin, G. T. Barradas, M. Ovsiannikow, J. Limmo, W. F. Thompson, Prevalence of emotions, mechanisms, and motives in music listening: A comparison of individualist and collectivist cultures. *Psychomusicology Music Mind Brain* **26**, 293 (2016).

7. S. Saarikallio, V. Alluri, J. Maksimainen, P. Toiviainen, Emotions of music listening in Finland and in India: Comparison of an individualistic and a collectivistic culture. *Psychol. Music* **49**, 989–1005 (2021).

8. C. Harte, M. Sandler, M. Gasser, Detecting harmonic change in musical audio in *Proceedings of the 1st ACM Workshop on Audio and Music Computing Multimedia*, (ACM, 2006), pp. 21–26.

9. O. Lartillot, T. Eerola, P. Toiviainen, J. Fornari, Multi-Feature Modeling of Pulse Clarity: Design, Validation and Optimization. in *ISMIR*, (2008), pp. 521–526.
